# The Interplay of Intrinsic and Extrinsic Bounded Noises in Biomolecular Networks

**DOI:** 10.1371/journal.pone.0051174

**Published:** 2013-02-21

**Authors:** Giulio Caravagna, Giancarlo Mauri, Alberto d'Onofrio

**Affiliations:** 1 Dipartimento di Informatica, Sistemistica e Comunicazione, Università degli Studi Milano-Bicocca, Milan, Italy; 2 Department of Experimental Oncology, European Institute of Oncology, Milan, Italy; Uni. of South Florida, United States of America

## Abstract

After being considered as a nuisance to be filtered out, it became recently clear that biochemical noise plays a complex role, often fully functional, for a biomolecular network. The influence of intrinsic and extrinsic noises on biomolecular networks has intensively been investigated in last ten years, though contributions on the co-presence of both are sparse. Extrinsic noise is usually modeled as an unbounded white or colored gaussian stochastic process, even though realistic stochastic perturbations are clearly bounded. In this paper we consider Gillespie-like stochastic models of nonlinear networks, i.e. the intrinsic noise, where the model jump rates are affected by colored bounded extrinsic noises synthesized by a suitable biochemical state-dependent Langevin system. These systems are described by a master equation, and a simulation algorithm to analyze them is derived. This new modeling paradigm should enlarge the class of systems amenable at modeling. We investigated the influence of both amplitude and autocorrelation time of a extrinsic Sine-Wiener noise on: 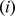 the Michaelis-Menten approximation of noisy enzymatic reactions, which we show to be applicable also in co-presence of both intrinsic and extrinsic noise, 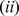 a model of enzymatic futile cycle and 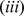 a genetic toggle switch. In 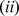 and 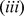 we show that the presence of a bounded extrinsic noise induces qualitative modifications in the probability densities of the involved chemicals, where new modes emerge, thus suggesting the possible functional role of bounded noises.

## Introduction

Cellular functions and decisions are implemented through the coordinate interactions of a very large number of molecular species. Central unit of these processes is the DNA, a polymer that is in part segmented in subunits, called genes, which control the production of the key cellular molecules: the proteins, via the mechanism of the transcription. Some relevant proteins, called transcription factors, in turn interact with genes to modulate either the production of other proteins or their own production.

Given the above rough outlook of the intracellular machineries it is not surprising that two modeling tools, actually born in other applicative domains, revealed to be of the utmost relevance in molecular biology. They are the inter-related concepts of feedback [1], [2] and of network [3]–[6], with their mathematical backbones: the dynamical systems theory and the graph theory, respectively. From the interplay and integration of these two theories with molecular biology, a new scientific field has appeared: Systems Biology [3]–[5].

Mimicking general chemistry, bipartite graphs were initially introduced in cellular biochemistry simply to formalize the informal diagrams representing biomolecular reactions [8]. Afterwards, and especially after the deciphering of genomes, it became clear that higher level concepts of network theories were naturally able to unleash fundamental biological properties, that were not previously understood. We briefly mention here the concepts of hub gene, and of biomolecular motif [3]–[7].

Note that the concept of network is also historically important in early phases of Systems Biology. Indeed, the first dynamical models in molecular biology were particular finite automata (graph-alike structures) called *boolean networks* [16]. These first pioneering investigations on the dynamics of biomolecular networks stressed two concepts that revealed nowadays to be two hallmarks in Systems Biology.

The first key concept is that biomolecular networks are multistable [12]–[15]. Indeed, it was quite soon understood – both experimentally and theoretically – that multiple locally stable equilibria allows for the presence of multiple functionalities, even in small groups of interplaying proteins [7], [17]–[25].

The second key concept is that the dynamic behavior of a network is never totally deterministic [9]–[11], but it exhibits more or less strong stochastic fluctuations due to its interplay with many, and mainly unknown, other networks, as well as with various random signals coming from the extracellular world. For long time the stochastic effects due these two classes of interactions were interpreted as a disturbance inducing undesired jumps between states or, with marginally functional role, as an external initial input directing towards one of the possible final states of the network in study. In any case, in the important scenario of deterministically monostable networks the stochastic behavior under the action of extrinsic noises was seen as unimodal. In other words, external stochastic effects were seen similarly as in radiophysics, namely as a disturbance more or less obfuscating the real signal, to be controlled by those pathways working as a low-pass analog filter [26], [27]. For these reasons, a number of theoretical and experimental investigations focused on the existence of noise-reducing sub-networks [26], [28], [29]. However, it has been recently shown the existence of fundamental limits on filtering noise [30].

Moreover, if noises were only pure nuisances, there would be an interesting consequence. Indeed, in such a case a monostable network in presence of noise should exhibit more or less large fluctuations around the unique deterministic equilibrium. In probabilistic languages this means that the probability distribution of the total signal (noise plus deterministic signal) should be a sort of “bell” centered more or less at the deterministic equilibrium, i.e. the probability distribution should be unimodal. However, at the end of seventies it became clear in statistical physics that the real stochastic scenario is far more complex, and the above-outlined correspondence between deterministic monostability and stochastic monomodality in presence of external noise was seriously challenged [31]. Indeed, it was shown that many systems that are monostable in absence of external stochastic noises have, in presence of random Gaussian disturbances, multimodal equilibrium probability densities. This counter-intuitive phenomenon was termed noise-induced transition [31], and it has been shown relevant also in genetic networks [32], [33].

Above we mainly focused on external random perturbations acting on genetic and other biomolecular networks. In the meantime, experimental studies revealed the other and equally important role of stochastic effects in biochemical networks by showing that many important transcription factors, as well as other proteins and mRNA, are present in cells with very low concentrations, i.e. with a small number of molecules [34]–[36]. Moreover, it was shown that RNA production is not continuous, but instead it has the characteristics of stochastic bursts [37]. Thus, a number of investigations has focused on this internal stochastic effect, the “intrinsic noise” as some authors term it [39], [40]. In particular, it was shown – both theoretically and experimentally – that also the intrinsic noise may induce multimodality in the discrete probability distribution of proteins [33], [41]. However, the fact that intrinsically stochastic systems may exhibit behaviors similar to systems affected by extrinsic Gaussian noises was very well known in statistical and chemical physics, where this was theoretically demonstrated by approximating the exact Chemical Master Equations with an appropriate Fokker-Planck equation [42]–[44], an approach leading to the Chemical Langevin Equation [45].

Thus, after that for some time noise was mostly seen as a nuisance, more recently it has finally been appreciated that the above-mentioned and other noise-related phenomena may in many cases have a constructive, functional role (see [46], [47] and references therein). For example, noise-induced multimodality allows a transcription network for reaching states that would not be accessible if the noise was absent [33], [46], [47]. Phenotype variability in cellular populations is probably the most important macroscopic effect of intracellular noise-induced multimodality [46].

In Systems Biology, from the modeling point of view Swain and coworkers [35] were among the first to study the co-presence of both intrinsic and extrinsic randomness, by stressing the synergic role in modifying the velocity and average in the context of the basic network for the production and consumption of a single protein, in absence of feedbacks. These and other important effects were shown, although nonlinear phenomena such as multimodality were absent. The above study is also remarkable since: 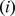 it has stressed the role of the autocorrelation time of the external noise and, differently from other investigations, 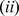 it has stressed that modeling the external noise by means of a Gaussian noise, either white or colored, may induce artifacts. In fact, since the perturbed parameters may become negative, the authors employed a lognormal positive noise to model the extrinsic perturbations. In particular, in [35] a noise obtained by exponentiating the classical Orenstin-Uhlenbeck noise was used [31].

From the data analysis point of view, You and collaborators [48] and Hilfinger and Paulsson [49] recently proposed interesting methodologies to infer by convolution the contributions of extrinsic noise also in some nonlinear networks, including a synthetic toggle switch [48].

Our aim here is to provide mathematical tools – and motivating biological examples – for the computational investigation of the co-presence of extrinsic and intrinsic randomness in nonlinear genetic (or in other biomolecular) networks, in the important case of not only non-Gaussian, but also bounded, external perturbations. We stress that, at the best of knowledge, this was never analyzed before. Indeed, by imposing a bounded extrinsic noise we increase the degree of realism of a model, since the external perturbations must not only preserve the positiveness of reaction rates, but must also be bounded. Moreover, it has also been shown in other contexts such as mathematical oncology [50]–[52] and statistical physics [50], [53]–[55] that: 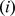 bounded noises deeply impact on the transitions from unimodal to multimodal probability distribution of state variables [51]–[55] and 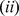 the dynamics of a system under bounded noise may be substantially different from the one of systems perturbed by other kinds of noises, for example there is dependence of the behavior on the initial conditions [51].

Here we assess the two most fundamental steps of this novel line of research.

The first step is to identify a suitable mathematical framework to represent mass-action biochemical networks perturbed by bounded noises (or simply left-bounded), which in turn can depend on the state of the system. To this extent, in the first part of this work we derive a master equation for these kinds of systems in terms of the differential Chapman-Kolgomorov equation (DCKE) [42], [56] and propose a combination of the Gillespie's Stochastic Simulation Algorithm (SSA) [38], [39] with a state-dependent Langevin system, affecting the model jump rates, to simulate these systems.

The second step relates to the possibility of extending, in this “doubly stochastic” context, the Michaelis-Menten Quasi Steady State approximation (QSSA) for enzymatic reactions [57]. We face the validity of the QSSA in presence of both types of noise in the second part of this work, where we numerically investigate the classical Enzyme-Substrate-Product network. The application of QSSA in this network has been recently investigated by Gillespie and coworkers in absence of extrinsic noise [58]. Based on our results, we propose the extension of the above structure also to more general networks than those ruled by the rigourous mass-action law via a stochastic QSSA.

Finally, we stress that the interplay between the extrinsic and intrinsic noises affecting a biomolecular network might impact on the dynamics of the involved molecules in many different and complex ways. As such, in our opinion this topic cannot be exhausted in a single work. For this reason, we provided three examples of interest in biology, and of quite different natures. One is the above-mentioned Michaelis-Menten reaction, the other two are illustrated in the third part of this work, and are the following: 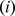 a futile cycle [33] and 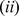 a genetic toggle switch [18], which is a fundamental motif for cellular differentiation and for other switching functions. As expected, the co-presence of both intrinsic stochasticity and bounded extrinsic random perturbations suggests the presence of possibly unknown functional roles for noise in both networks. The described noise-induced phenomena are shown to be strongly related to physical characteristics of the extrinsic noise such as the noise amplitude and its autocorrelation time.

## Methods

### Noise-free stochastic chemically reacting systems

We start by recalling the Chemical Master Equation and the Stochastic Simulation Algorithm (SSA) by Doob and Gillespie [38], [39]. Systems where the jump rates are time-constant are hereby referred to as stochastic noise-free systems. We consider a well stirred system of molecules belonging to 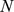 chemical species 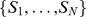 interacting through 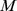 chemical reactions 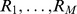. We represent the (discrete) state of the target system with a 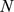-dimensional integer-valued vector 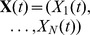 where 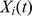 is the number of molecules of species 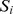 at time 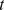. To each reaction 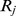 is associated its stoichiometric vector 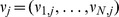, where 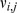 is the change in the 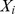 due to one 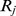 reaction. The stoichiometric vectors form the 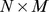 stoichiometry matrix 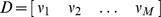. Thus, given 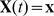 the firing of reaction 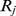 yields the new state 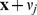. A propensity function 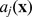 [38], [39] is associated to each 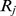 so that 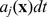, given 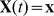, is the probability of reaction 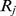 to fire in state 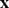 in the infinitesimal interval 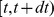. Table 1 summarizes the analytical form of such functions [38]. For more generic form of the propensity functions (e.g. Michaelis-Menten, Hill kinetics) we refer to [62].

**Table 1:** Gillespie propensity functions. Analytical form of the propensity functions [38].

Order	Reaction	Propensity
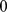 -th	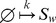	*k*
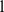 -st	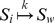	*kX_i_*(*t*)
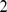 -nd	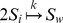	*kX_i_*(*t*)(*X_i_*(*t*)−1)/2
	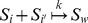	*kX_i_*(*t*)(*X_i_*_′_(*t*)

We recall the definition of the *Chemical Master Equation* (CME) [38], [39], [60], [61] describing the time-evolution of the probability of a system to occupy each one of a set of states. We study the time-evolution of 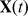, assuming that the system was initially in some state 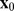 at time 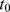, i.e. 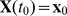. We denote with 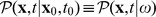 the probability that, given 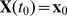, at time 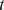 it is 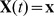. From the usual hypothesis that at most one reaction fires in the infinitesimal interval 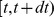, it follows that the time-evolution of 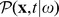 is given by the following partial differential equation termed “master equation”
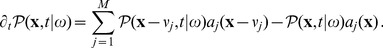
(1)


The CME is a special case of the more general Kolmogorov Equations [63], i.e. the differential equations corresponding to the time-evolution of stochastic Markov jump processes. As it is well known, the CME can be solved analytically only for a very few simple systems, and normalization techniques are sometimes adopted to provide approximate solutions [64]. However, algorithmic realization of the process associated to the CME are possible by using the Doob-Gillespie Stochastic Simulation Algorithm (SSA) [38], [39], [60], [61], summarized as Algorithm 1 (Table 2). The SSA is reliable since it generates an *exact* trajectory of the underlying process. Although equivalent formulations exist [38], [39], [65], as well as some approximations [62], [66], [67], here we consider its *Direct Method* formulation without loss of generality.

**Table 2:** Algorithm 1 Gillespie Stochastic Simulation Algorithm [38], [39].

1: **Input**: initial time *t*_0_, state **x**_0_ and final time *T*;
2: set **x**←**x**_0_ and *t*←*t*_0_;
3: **while** *t*<*T* **do**
4: define 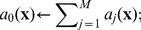
5: let 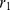 , *r*_2_∼*U* [0,1];
6: determine next jump as 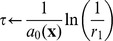 ;
7: determine next reaction as 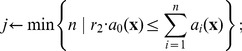
8: set **x**←**x**+*ν_j_* and *t*←*t*+*τ*;
9: **end while**

The SSA is a dynamic Monte-Carlo method describing a statistically correct trajectory of a discrete non-linear Markov process, whose probability density function is the solution of equation (1) [68]. The SSA computes a single realization of the process 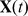, starting from state 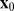 at time 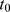 and up to time 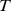. Given 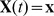 the putative time 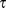 for the next reaction to fire is chosen by sampling an exponentially distributed random variable, i.e. 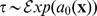 where 
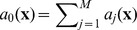
 and 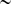 denotes the equality in law between random variables. The reaction to fire 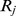 is chosen with weighted probability 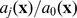, and the system state is updated accordingly.

The correctness of the SSA comes from the relation between the jump process and the CME [38], [68]. In fact, the probability, given 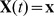, that the next reaction in the system occurs in the infinitesimal time interval 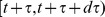, denoted 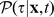, follows
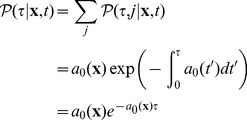
(2)since 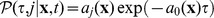 is the probability distribution of the putative time for the next firing of 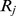, and the formula follows by the independency of the reaction firings. Notice that in equation (2) 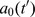 represents the propensity functions evaluated in the system state at time 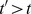, i.e. as if they were time-dependent functions. In the case of noise-free systems that term evaluates as 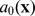 for any 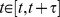, i.e. it is indeed time-homogenous whereas in more general cases it may not, as we shall discuss later. Finally, the probability of the reaction to fire at 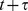 to be 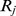 follows by conditioning on 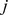, that is
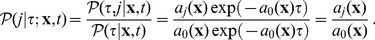
(3)


### Noisy stochastic chemically reacting systems

We now introduce a theory of stochastic chemically reacting systems with bounded noises in the jump rates by combining *Stochastic Differential Equations* and the SSA. Here we consider a system where each propensity function may be affected by a *extrinsic* noise term. In general, such a term can be either a time or state-dependent function, and the propensity function for reaction 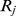 reads now as
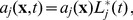
(4)where 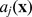 is a propensity function of a type listed in Table 1. The noisy perturbation term 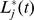 is positive and bounded by some 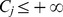, i.e.
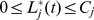
(5)so we are actually considering both bounded and right-unbounded noises, i.e. 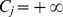. In the former case we say that the 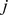-th extrinsic noise is bounded, in the latter that it is left-bounded.

Note that in applications we shall mainly consider unitary mean perturbations, that is
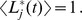



We consider here that the extrinsic noisy disturbance 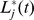 is a function of a more generic 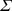-dimensional noise 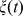 with 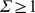 so we write 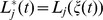 and equation (4) reads as
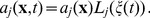
(6)


Notice that the use of a vector in equation (6) provides the important case of multiple reactions sharing the same noise term, i.e. the reactions may be affected in the same way by a unique noise source.

In equation (6) 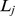 is a continuous functions 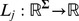 and 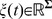 is a colored and, in general, non-gaussian noise that may depend on the state 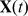 of the chemical system. The dynamics of 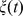 is described by a 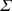-dimensional Langevin system
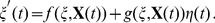
(7)


Here, 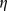 is a 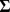-dimensional vector of uncorrelated white noises of unitary intensities, 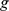 is a 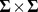 matrix which we shall mainly consider the be diagonal and 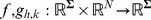.

When 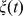 does not directly depend on 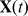, i.e. the extrinsic noise depends on an external source, which is the kind of noise we mainly consider, equation (7) reduces to
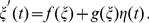
(8)


We stress that the “complete” Langevin system in equation (7) is not a mere analytical exercise, but it has the aim of phenomenologically modeling extrinsic noises that are not totally independent of the process in study.

#### The Chapman-Kolmogorov Forward Equation

When a discrete-state jump process as one of those described in previous section is linked with a continuous noise the state of the stochastic process is the vector
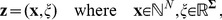
(9)and the state space of the process is now 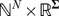. Our total process can be considered as a particular case of the general Markov process where diffusion, drift and discrete finite jumps are all co-present for all state variables [42], [56]. For this very general family of stochastic processes the dynamics of the probability of being in some state 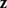 at time 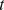, given an initial state 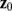 at time 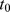 shortly denoted as 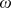, is described by the *differential Chapman-Kolgomorov equation* (DCKE) [42], [56], whose generic form is



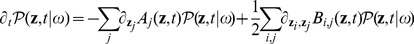
(10)

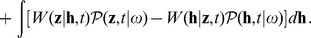



Here 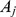 forms the drift vector for 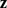, 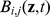 the diffusion matrix and 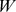 the jump probability. For an elegant derivation of the DCKE from the integral Chapman-Kolgomorov equation [63] we refer to [56]. This equation describes various systems, in fact we remind that 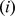 the Fokker-Planck equation is a particular case of the DCKE without jumps (i.e. 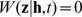), 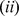 the CME in equation (1) is the DCKE without brownian motion and drift (i.e. 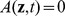 and 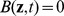), 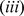 the Liouville equation is the DCKE without brownian motion and jumps (i.e. 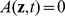 and 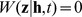) and 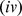 the ODE with jumps correspond to the case where only diffusion is absent (i.e. 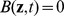).

We stress that, at the best of our knowledge, this is the first time where a master equation for stochastic chemically reacting systems combined with bounded noises is considered. Let
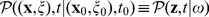
(11)be the probability that at time 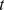 it is 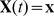 and 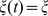, given 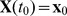 and 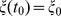. The time-evolution of 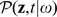 is equation (10) where drift and diffusion are given by the Langevin equation (7), that is
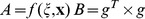
(12)with 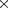 the standard vector multiplication and 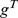 the transpose of 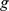. Moreover, since only finite jumps are possible, then the jump functions and diffusion satisfy
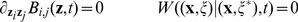
(13)for any 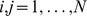, and noise 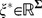. Summarizing, for the systems we consider the DCKE in equation (10) reads as



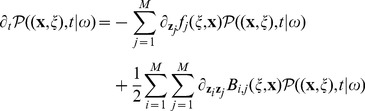
(14)

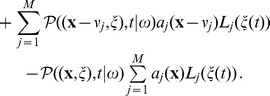



This equation is the natural generalization of the CME in equation (1), and completely characterize noisy systems. As such, however, its realization can be prohibitively difficult and is hence convenient to define algorithms to perform the simulation of noisy systems.

#### The SSA with Bounded Noise

We now define the *Stochastic Simulation Algorithm with Bounded Noise* (SSAn). The algorithm performs a realization of the stochastic process underlying the system where a (generic) realization of the noise is assumed. As for the CME and the SSA, this corresponds to computing a realization of a process satisfying equation (14). This implies that, as for the SSA, the SSAn is reliable since the generated trajectory is *exact*. This, in future, will allow to use the SSAn as a base to define approximate simulation to sample from equation (14), as it is done from the SSA and the CME [62], [66], [67]. The SSAn takes inspiration from the (generic) SSA with time-dependent propensity functions [69] as well as the SSA for hybrid deterministic/stochastic systems [70]–[73], thus generalizing the jump equation (2) to a time inhomogeneous distribution, which we discuss in the following.

For a system with 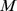 reactions the time evolution equation for 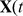) is
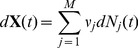
(15)where 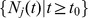 is the stochastic process counting the number of times that 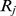 occurs in 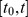 with initial condition 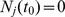. For Markov processes 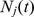 is an inhomogeneous Poisson process satisfying
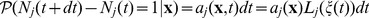
(16)when 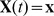. In hybrid systems this is is a doubly stochastic Poisson process with time-dependent intensity, in our case this is a Cox process [74], [75] since the intensity itself is a stochastic process, i.e. it depends on the stochastic noise. More simply, in noise-free systems, this equation evaluates as 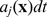, thus denoting a time homogeneous Poisson process. As in [70], [72], [73], [76], [77] such a process ca be transformed in a time homogenous Poisson process with parameter 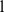, and a simulation algorithm can be exploited. Let us denote with 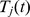 the time at next occurrence of reaction 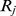 after time 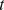, then
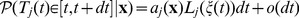
(17)follows by equation (16) and higher order terms vanish by the usual hypothesis that the reaction firings are locally independent, as in the derivation of equation (1). Given the system to be in state 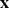 at time 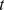, the transformation
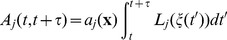
(18)which is a monotonic (increasing) function of 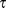 is used to determine the putative time for 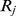 to fire. Given a sequence 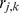 of independent exponential random variables with mean 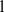 for 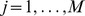 and 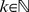, equation (16) implies that



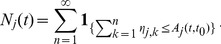
(19)This provides that, if the systems is in state 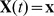, then the next time for the next reaction firing of 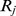 is the smallest time 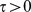 such that
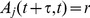
(20)with 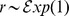, and thus the next jump of the overall system is taken as the minimum among all possible times, that is by solving equality
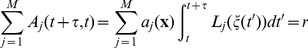
(21)with 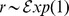. This holds because 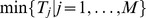 is still exponential with parameter 
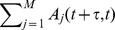
 and the jumps are independent. We remark that for a noise-free reaction 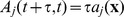, thus suggesting that the combination of noisy and noise-free reactions is straightforward. The index of the reaction to fire is instead a random variable following



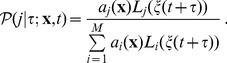
(22)The SSAn is Algorithm 2 (Table 3); its skeleton is similar to Gillespie's SSA, so the algorithm simulates the firing of 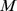 reactions in a (discrete) state 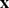 tracking molecule counts. In addiction to the SSA, this algorithm also tracks the (continuos) state storing the noises.

**Table 3:** Algorithm 2 Stochastic Simulation Algorithm with Bounded Noises (SSAn).

1: **Input**: initial time *t*_0_, state **x**_0_ and final time*T*;
2: set **x**←**x**_0_ and *t*←*t*_0_;
3: **while** *t*<*T* **do**
4: let 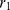 , *r*_2_∼*U* [0,1];
5: find next jump by solving equation (21), that is 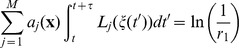 while generating noise *ξ*(*t*) in *t*′ε[*t*, *t*+*τ*];
6: determine next reaction as 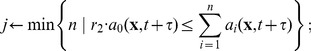
7: set **x**←**x**+*ν_j_* and *t*←*t*+*τ*;
8: **end while**

As for the SSA, jumps are determining by using two uniform numbers 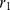 and 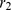. Step 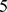 is the (joint) solution of both equation (21) and Langevin system (7), i.e. 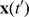 in 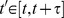. This allows to both 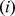 determine the putative time for the next reaction to fire, i.e. the 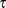 solving equation (21), and to 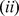 update noise realization, i.e. system (7). This step is the computational bottleneck of this algorithm since it can not be analytical, unless for simple cases, as instead was for the SSA (step 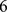 had an exact solution for 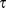). We remark that this does not affect the exactness of the SSAn with respect to the trajectory of the underlying stochastic process. Being non-analytical an iterative method, e.g. the Newton-Raphson, has to be embedded in the SSAn implementation. Furthermore, noise integration is also non-analytical thus inducing a further numerical approximation issue. To this extent, the integral in equation (21), i.e. a conventional Lebesgue integral since the perturbation 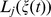 is a colored stochastic process [78], can be solved by adopting an interpolation scheme. An example linear scheme is
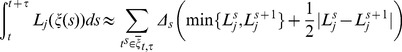
(23)where
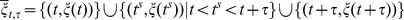
(24)is a single trajectory of the vectorial noise process in 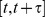, 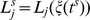 for 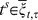 and 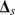 the noise granularity. We remark that this is a discretization of a continuous noise, thus inducing an approximation, but is in general the only possible approach. To reduce approximation errors in the SSAn the maximum size of the jump in the noise realization, i.e. the noise granularity 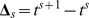, should be much smaller than the minimum autocorrelation time of the perturbing stochastic processes 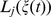.

Once the jumpt time 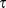 has been determined, sample values for 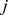 are determined according to equation (22) in step 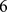, as similarly done in the SSA. This sample is again numerical and an arbitrary precision can be obtained by properly generating the noise.

All these two equations, as well as the numerical method to solve equation (21) are implemented in the SSAn implementation which can be found in the NoisySIM free library [59], as discussed in the Results section.

#### Extension to non mass-action nonlinear kinetic laws

Large networks with large chemical concentrations, i.e. characterized by deterministic behaviors, are amenable to significant simplifications by means of the well known *Quasi Steady State Approximation* (QSSA) [7], [57], [58], [79]. The validity conditions underlying these assumptions are very well-known in the context of deterministic models [57], despite not much being known for the corresponding stochastic models. Recently, Gillespie and coworkers [58] showed that, in the classical Michaelis-Menten Enzyme-Substrate-Product network, a kind of *Stochastic QSSA* (SQSSA) may be applied as well, and that in such its limitations are identical to the deterministic QSSA. Thus, it is of interest to consider SQSSAs also in our “doubly stochastic” setting, even though possible pitfalls may arise due to the presence of the extrinsic noises. As an example, in Results section we will present numerical experiments similar to those of [58], with the purpose of validating the SQSSA for noisy Michaelis-Menten enzymatic reactions.

Of course, in a SQSSA not only the propensities may be nonlinear function of state variables, but they may depend nonlinearly also on the perturbations, so that instead of the elementary perturbed propensities we shall have generalized perturbed propensities of the form
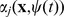
where 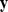 is a vector with elements 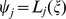 for 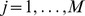. This makes possible, within the above outlined limitation for the applicability of the SQSSA, to write a DCKE for these systems as



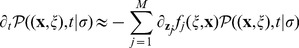
(25)


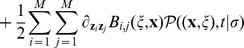


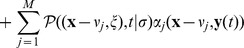


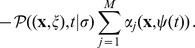



As far as the simulation algorithm is concerned, it remains quite close to Algorithm 2 (Table 3) provided that the jump times are sampled according to the following distribution
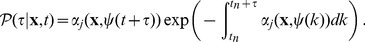
(26)


## Results

We performed SSAn-based analysis of some simple biological networks, actually present in most complex realistic networks. We start by studying the legitimacy of the stochastic Michaelis-Menten approximation of when noise affects enzyme kinetics [58]. Then we study the role of the co-presence of intrinsic and extrinsic bounded noises in a in a model of enzymatic futile cycle [33] and, finally, in a bistable “toggle switch” model of gene expression [24], [86]. All the simulations have been performed by a Java implementation of the SSAn, currently available within the NoisySIM free library [59].

### The Sine-Wiener noise [53]

The bounded noise 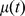 that we use in our simulations is obtained by applying a bounded continuous function 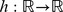 to a random walk 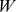, i.e. 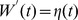 with 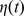 a white noise. We have
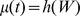
so that for some 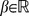 it holds 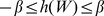. The effect of the truncation of the tails induced by the approach here illustrated is that, due to this “compression”, the stationary probability densities of this class of processes satisfy



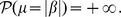



Probably the best studied bounded stochastic process obtained by using this approach is the so-called Sine-Wiener noise [53], that is
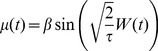
(27)where 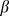 is the *noise intensity* and 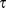 is the *autocorrelation time*. The average and the variance of this noise are



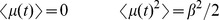
and its autocorrelation is such that [53]



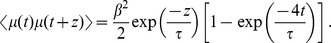



Note that, since we mean to use noises of the form 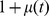, i.e. the unitary-mean perturbations in equation (6), then the noise amplitude must be such that 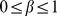.

For this noise, the probability density is the following [89]

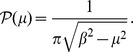



By these properties, this noise can be considered a realistic extension of the well-known symmetric dichotomous Markov noise 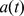, whose stationary density is 
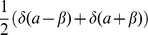
, for 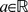 and 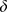 the Dirac delta function [80]. Finally, we remark that the white-noise process 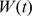 is generated at times 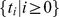 according to the recursive schema 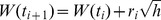 with initial condition 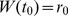. Here 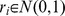 and 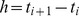 for 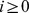 is its *discretization step*; it has to satisfy 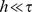 so we typically chose 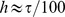. Notice that the noise autocorrelation is expected to deeply impact on the simulation times.

### Enzyme kinetics

Enzyme-catalyzed reactions are fundamental for life, and in deterministic chemical kinetics theories are often conveniently represented in an approximated non mass-action form, the well-known Michaelis-Menten kinetics [7], [57], [58]. Such approximation of the exact mass-action model is based on a Quasi Steady-State Assumption (QSSA) [57], [79], valid under some well known conditions. In [58] it is studied the legitimacy of the Michaelis-Menten approximation of the Enzyme-Substrate-Product stochastic reaction kinetics. Most important, it is shown that such a stochastic approximation, i.e. the SQSSA in previous section, obeys the same validity conditions for the deterministic regime. This suggests the legitimacy of using – in case of low number of molecules – the Gillespie algorithm not only for simulating mass-action law kinetics, but more in general to simulate more complex rate laws, once a simple conversion of deterministic Michaelis-Menten models is performed and provided – of course – that the SQSSA validity conditions are fulfilled.

In this section we investigate numerically whether the Michaelis-Menten approximations and the stochastic results obtained in [58] still hold true in case that a bounded stochastic noise perturb the kinetic constants of the propensities of the exact mass-action law system Enzyme-Substrate-Product. Let 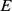 be an enzyme, 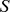 a substrate and 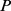 a product, the exact mass-action model of enzymatic reactions comprises the following three reactions
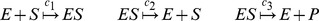
where 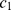, 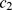 and 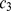 are the kinetic constants. The network describes the transformation of substrate 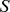 into product 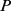, as driven by the formation of the enzyme-substrate complex 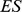, which is reversible.

The deterministic version of such reactions is
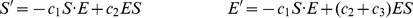
(28)

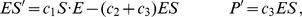
where we write 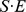 to distinguish the multiplication of 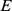 and 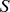 from complex 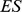. By the relations
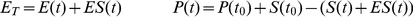
(29)a QSSA reduces to one the number of involved equations. Indeed, since 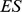 is in quasi-steady-state, i.e. 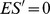, then



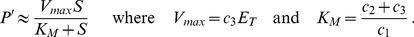
(30)Here 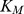 is termed the Michaelis-Menten constant. In practice, the QSSA permits to reduce the three-reactions model to the single-reaction model
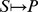
with non mass-action non linear rate 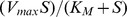. In [58] the condition
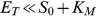
(31)is used to determine a region of the parameters space guaranteeing the legitimacy of the Michaelis-Menten approximation. When condition (31) holds, a separation exists between the fast pre-steady-state and the slower steady-state timescales [79] and the solution of the Michaelis-Menten approximation closely tracks the solution of the exact model on the slow timescale.

**Figure 1:**
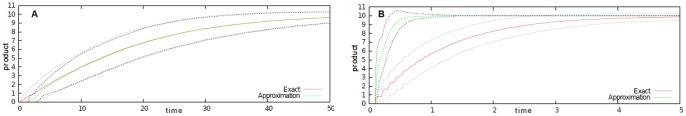
Noise-free Enzyme-Substrate-Product system. Product formation (averages of 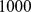 simulations, plotted with dotted standard deviation) for both exact and approximated Michaelis-Menten kinetics. We have set 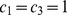 and 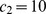; the initial configuration is 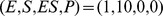 in **A** and 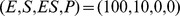 in **B**.

Here we show that the same condition is sufficient to legitimate the Michaelis-Menten approximation with bounded noises arbitrarily applied to any of the involved reactions. We start by recalling the result in [58] about the noise-free models given in Table 4. We considered two initial conditions: 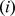 one with 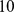 copies of substrate, 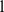 enzyme and 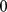 complexes and products, and 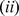 one with 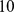 copies of substrate, 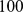 enzyme and 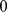 complexes and products. As in [58] we set 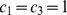 and 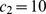; notice that the parameters are dimensionless and, more important, in 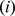 they satisfy condition (31) since 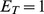 and 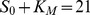, in 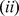 no. In Figure 1 we reproduced the results in [58] for 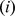 in right panel and 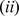 in left. As expected, in 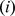 the approximation is valid on the slow time-scale, and not valid in the fast, i.e. for 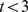, in 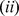 it is not valid also in the slow time-scale.

**Table 4:** Enzyme-Substrate-Product model.

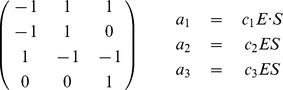
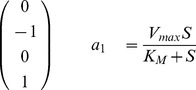

Exact model (left) and Michaelis-Menten approximation (right) of enzymatic reactions: the stoichiometry matrixes (rows in order 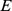, 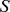, 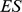, 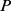) and the propensity functions.

If noises are considered the models in Table 4 change accordingly. So, for instance when independent Sine-Wiener noises are applied to each reaction, the exact model becomes
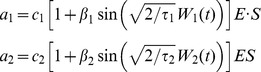


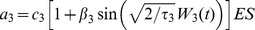
and the Michaelis-Menten constant becomes the time-dependent function



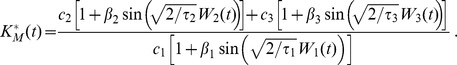



Notice that the nonlinear approximated propensity 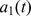 is now time-dependent, and, moreover, it depends nonlinearly on the noises affecting the system.

Thus condition (31) becomes time-dependent and we rephrase it to be
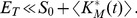
(32)


Note that if 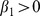 then 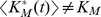, whereas if 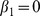 then 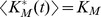.

**Figure 2:**
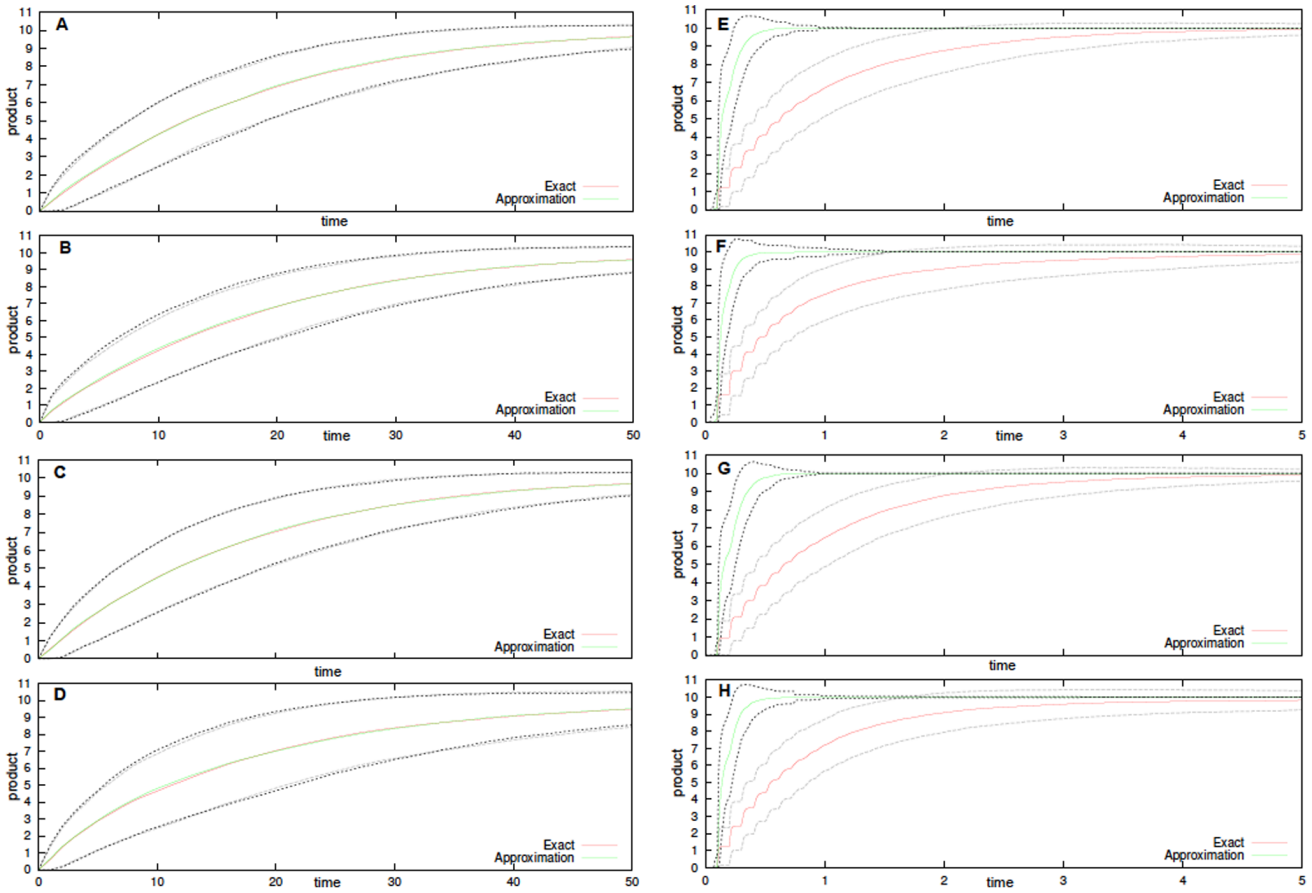
Stochastically perturbed Enzyme-Substrate-Product system. Product formation (averages of 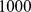 simulations, plotted with dotted standard deviation) for both exact and approximated Michaelis-Menten kinetics. In **A**, **B**, **C** and **D** the initial configuration is 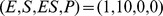, in all other panels is 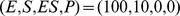. Independent Sine-Wiener noises are present in all the reactions. For 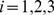, 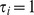 in **A**, **B**, **E** and **F**, and 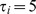 in all other panels. Also, 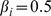 in **A**, **C**, **E** and **G**, and 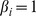 in all other panels.

Each of the shown figures is the result of 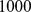 simulations for model configuration where the simulation times, which span from few seconds to few minutes, depend on the noise correlation. When the same system of Figure 1 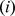 is extended with these noises the approximation is still valid, as shown in the top panels of Figure 2. In addition, the approximation is not valid when condition (32) does not hold, as shown in the bottom panels of Figure 2, as it was in Figure 1 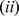. Notice that in there we use two different noise correlations, i.e. 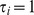 in the left and 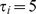 for 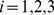 in the right column panels, thus mimicking noise sources with quite different characteristic kinetics. Also, we set two different noise intensities, i.e. 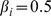 in top panels and 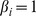 (maximum intensity) in bottom panels, whereas all the other parameters are as in Figure 1. Summarizing, we get a complete agreement between enzymatic reactions with/without noise, independently on the noise characteristics when it affects all of the reactions.

To strengthen this conclusion it becomes important to investigate whether it still holds when noises affects only a portion of the network and, also, whether it holds on the fast time-scale.

**Figure 3:**
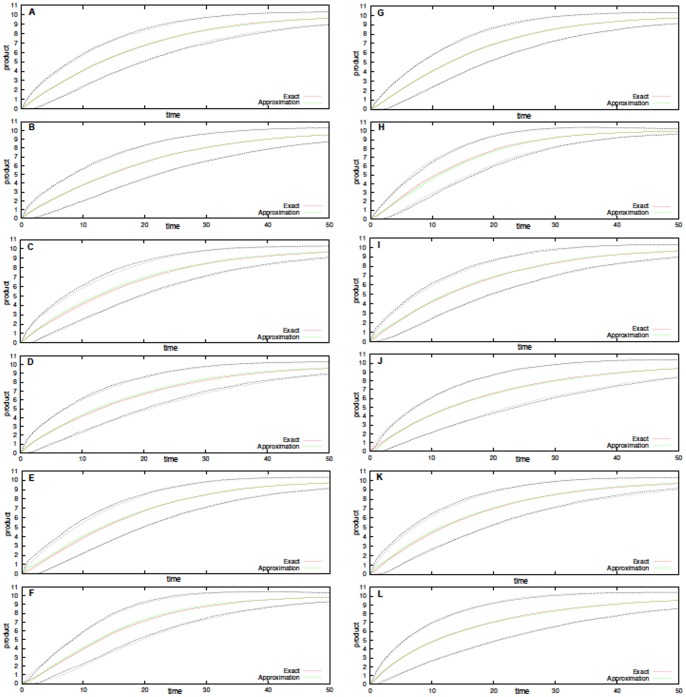
Stochastically perturbed Enzyme-Substrate-Product system. Product formation (averages of 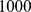 simulations, plotted with dotted standard deviation) for both exact and approximated Michaelis-Menten kinetics. In all panels the initial configuration is 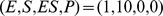. Here single Sine-Wiener noises various intensities and autocorrelations are used. In **A** 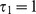 and 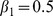, in **B** 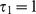 and 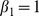, in **C** 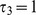 and 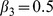, in **D** 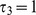 and 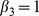, in **E** 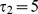 and 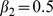, in **F** 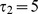 and 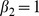, in **G** 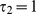 and 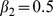, in **H** 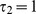 and 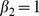, in **I** 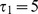 and 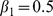, in **J** 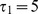 and 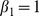, in **K** 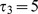 and 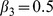 and in **L** 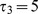 and 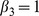. All other parameters are 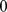.

As far as the number of noises is concerned, we investigated various single-noise configurations in Figure 3. In there we used a single noise, i.e. two out of the three noises have 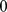 intensity, with both low and high intensities, i.e. 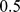 and 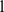. Also, in that figure we vary the noise correlation time as 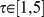. As hoped, the simulations show that the approximation is legitimate in the slow time-scale for all the various parameter configurations, thus independently on the presence of single or multiple noises.

**Figure 4:**
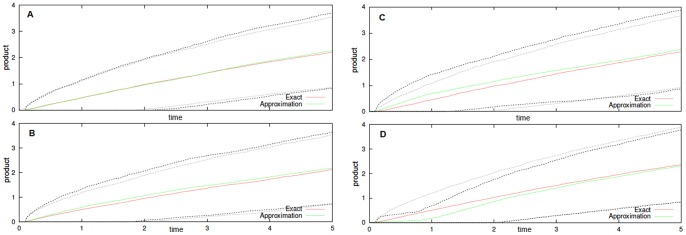
Stochastically perturbed Enzyme-Substrate-Product system. Product formation (averages of 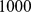 simulations, plotted with dotted standard deviation) for both exact and approximated Michaelis-Menten kinetics in the fast time-scale 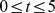. In all panels the initial configuration is 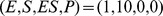. Here a single Sine-Wiener noise affects complex formation. In **A** 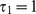 and 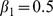, in **B** 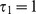 and 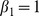, in **C** 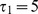 and 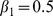, in **D** 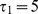 and 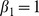.

Finally, as far as the legitimacy of the approximation in the fast time-scale is concerned, i.e. 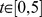, our simulations show a result of interest: if the noise correlation is small compared to the reference fast time-scale and if single noises are considered the noisy Michaelis-Menten approximation performs well also on the fast time-scale. We remark that this was not the case for the analogous noise-free scenario in Figure 1 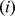. In support of this we plot in Figure 4 the fast time-scale for 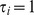 and 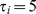 for the single noise model with a noise in the enzyme-substrate complex formation, i.e. 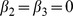. Similar evidences were found in the configurations plotted in Figure 3 (not shown).

### Futile cycles

In this section we consider a model of *futile cycle*, as the one computationally studied in [33]. The model consists of the following mass-action reactions
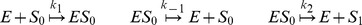


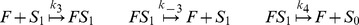
where 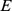 and 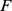 are enzymes, 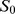 and 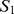 substrate molecules, and 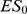 and 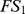 the complexes enzyme-substrate. Futile cycles are an unbiquitous class of biochemical reactions, acing as a motif in many signal transduction pathways [81].

Experimental evidences related the presence of enzymatic cycles with bimodalities in stochastic chemical activities [82]. As already seen in the previous section, Michaelis-Menten kinetics is not sufficient to describe such complex behaviors, and further enzymatic processes are often introduced to induce more complex behaviors. For instance, in deterministic models of enzymatic reactions feedbacks are necessary to induce bifurcations and oscillations. Instead, in [33] it is shown that, although the deterministic version of the model has a unique and attractive equilibrium state, stochastic fluctuations in the total number of 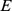 molecules may induce a transition from a unimodal to a bimodal behavior of the chemicals. This phenomenon was shown both by the analytical study of a continuous SDE model where the random fluctuations in the total number of enzyme 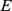 (both free and as a complex with 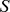) is modeled by means of a white gaussian noise on the one hand, and in a totally stochastic setting on the other hand. In the latter case it was assumed the presence of a third molecule 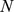 interacting with enzyme 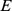 according to the following reactions
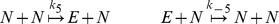


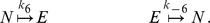



By using 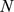 the stochastic model results to be both quantitatively and qualitatively different from the deterministic equivalent. These differences serve to confer additional functional modalities on the enzymatic futile cycle mechanism that include stochastic amplification and signaling, the characteristics of which depend on the noise.

Our aim here is to investigate whether bounded noises affecting the kinetic constant, and thus not modifying the topology of the futile cycle network, may as well induce transition to bimodality in the system behavior. To this aim, here we analyze three model configurations: 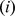 the noise-free futile cycle, namely only the first six reactions, 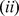 the futile cycle with the external noise as given by 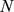 and 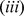 the futile cycle with a bounded noise on the binding of 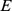 and 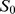, i.e. the formation of 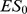, and 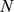 is absent.

In Table 5 the noise-free futile cycle is given as a stoichiometry matrix and 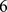 mass-action reactions. The model simulated in [33] is obtained by extending the model in the table with a stoichiometry matrix containing 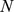 and four more mass-action reactions. For the sake of shortening the presentation we omit to show them here. The model with a bounded noise in 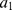 is obtained by defining

**Table 5:** Futile cycle model.

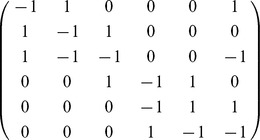	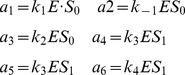

The noise-free enzymatic futile cycle [33]: the stoichiometry matrix (rows in order 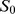, 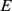, 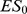, 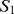, 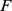, 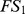) and the propensity functions.



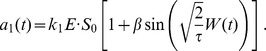



We simulated the above three models according to the initial condition used in [33] 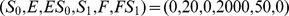 which is extended to account for 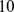 initial molecules of 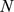, when necessary. The kinetic parameters are dimensionless and defined as 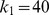, 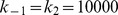, 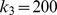, 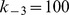, 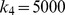 for the noise-free and the bounded noise case, and 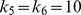, 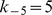 and 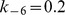 when the unimodal noise is considered [33]. Furthermore, when the bounded noise is considered the autocorrelation is chosen as 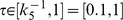 according to the highest rate of the reactions generating the unimodal noise.

**Figure 5:**
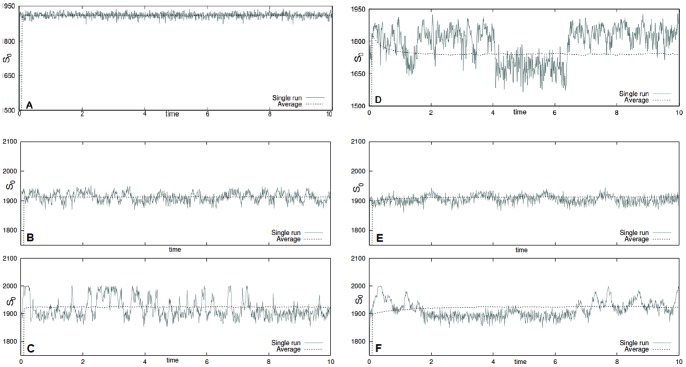
Stochastic models of futile cycles. Single run and averages of 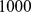 simulations for substrate 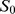 of the futile cycle models. In panel **A** the noise-free futile cycle and in panel **D** the extended noise-free model including the additional species 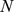. In bottom plots the cycle affected by bounded Sine-Wiener noise with: in **B** 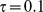 and 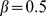, in **C** 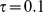 and 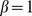, in **E** 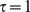 and 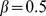, in **F** 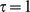 and 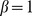. The initial configuration is always 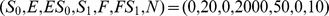; the kinetic parameters are 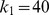, 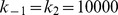, 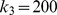, 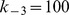 and 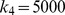 (noise-free and the bounded noise case), and 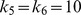, 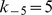 and 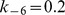 [33].

**Figure 6:**
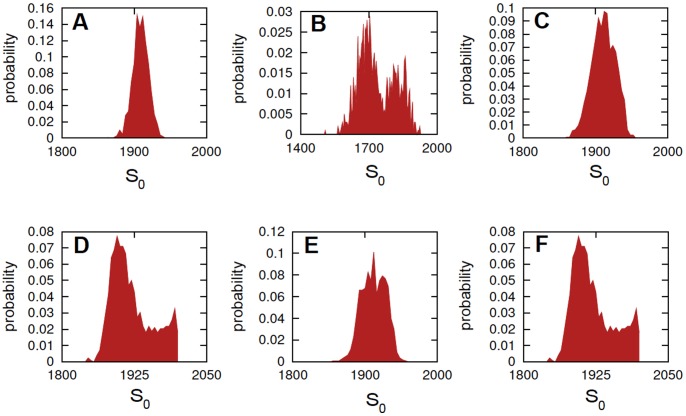
Stochastic models of futile cycles. Empirical probability density function for 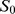 at 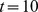 after 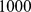 simulations for the futile cycle models with the parameter configurations considered in Figure 5. In panel **A** the noise-free cycle, in **B** the cycle affected by sine-Wiener noise with 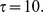 and 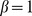, in **C** the noise-free modified cycle including the additional species 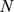. In bottom panels the cycle affected by sine-Wiener noise with: in **D** 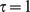 and 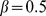, in **E** 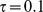 and 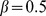 and in **F** 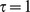 and 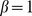.

In Figure 5 a single run and averages of 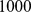 simulations for the futile cycle models are shown. In this case the simulation times span in range from 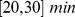 to 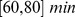, thus making the choice of good parameters more crucial than in the other cases. In Figure 5 the substrate 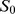 is plotted, and 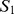 behaves complementarily. In top panels the noise-free (top) and the cycle unimodal noise as 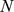 (bottom). In bottom panels the cycle with bounded noise and autocorrelation 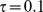 in (left) and 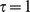 in (right). In both cases in the top panel the noise intensity is 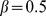 (top) and 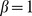 (bottom). The initial configuration is always 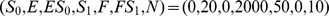 and the kinetic parameters are 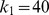, 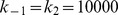, 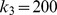, 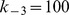, 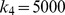 for the noise-free and the bounded noise case, and 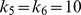, 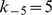 and 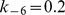 [33]. We also show in Figure 6 the empirical probability density function for the concentration of 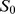, i.e. 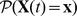 given the considered initial configuration, at 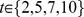 after 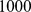 simulations for the futile cycle models with the parameter configurations considered in Figure 5. The analysis of such distributions outline that for the noise-free system the distributions are clearly unimodal, whereas for noisy futile cycle, in both cases, they are bi-modal. Moreover, it is important to notice that the smallest peak of the distribution, i.e. the rightmost, has a bigger variance when 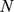 is considered, rather than when a bounded noise is considered.

### Bistable kinetics of gene expression

Let us consider a model by Zhdanov [24], [86] where two genes 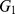 and 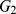, two RNAs 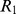 and 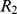 and two proteins 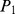 and 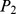 are considered. In such a model synthesis and degradation correspond to
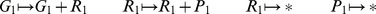


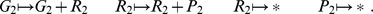



Such a reaction scheme is a genetic toggle switch if the formation of 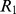 and 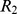 is suppressed by 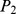 and 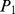, respectively [18], [25], [83]–[85]. Zhdanov further simplifies the schema by considering kinetically equivalent genes, and by assuming that the mRNA synthesis occurs only if 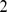 regulatory sites of either 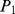 or 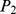 are free. The deterministic model of the simplified switch when synthesis is perturbed is
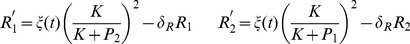
(33)

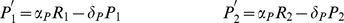
where the perturbation is



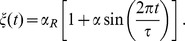



Here 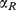, 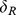, 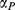 and 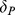 are the rate constants of the reactions involved, term 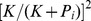 is the probability that 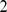 regulatory sites are free and 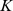 is the association constant for protein 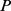. Notice that here perturbations are given in terms of a time-dependent kinetic function for synthesis, rather than a stochastic differential equation. Before introducing a realistic noise in spite of a perturbation we perform some analysis of this model. As in [86] we re-setted model (33) in a stochastic framework by defining the reactions described in Table 6. Notice that in there two reactions have a time-dependent propensity function, i.e. 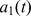 and 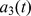 modeling synthesis.

**Table 6:** Toggle switch model.

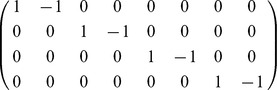	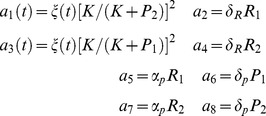

The bistable model of gene expression in [86]: the stoichiometry matrix (rows in order 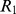, 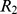, 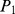, 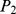) and the propensity functions.

**Figure 7:**
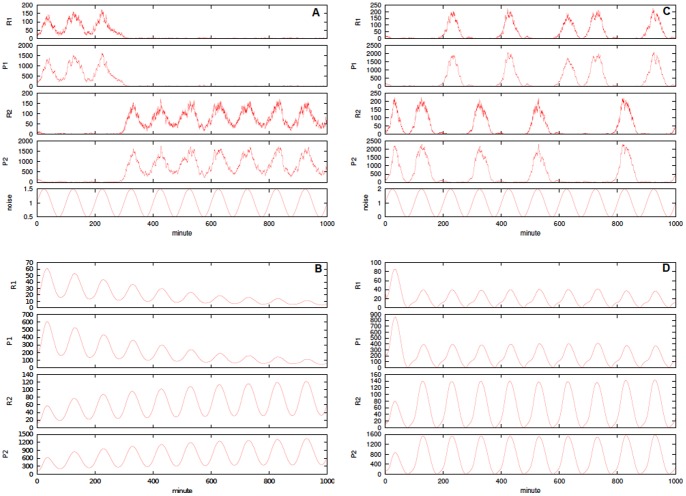
Periodically perturbed toggle switch. In the top panels a single run for Zhdanov model (33) with 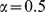 (**A**) and 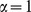 (**C**). In bottom plots averages of 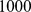 simulations are shown with 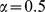 (**B**) and 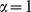 (**D**). In all cases 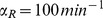, 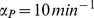, 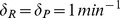, 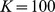 and 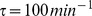 and the initial configuration is 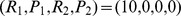. The noise realization is plotted for the single runs.

In the top panels of Figure 7 we show single runs for Zhdanov model where simulations are performed with the exact SSA with time-dependent propensity function. In [86] an exact SSA [39] is used to simulated the model under the assumption that variations in the propensity functions are slow between two stochastic jumps. This is true for 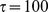 as in [86], but not true in general for small values of 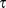. We considered an initial configuration with only 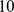 RNAs 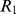. As in [86] we set 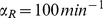, 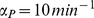, 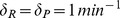, 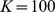 and 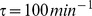; notice that this parameters are realistic since, for instance, protein and mRNA degradation usually occur on the minute time-scale [87]. We considered two possible noise intensities, i.e. 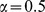 in left and 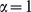 in right and, as expected, when 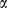 increases the number of switches increases. To investigate more in-depth this model we performed 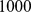 simulations for both the configurations. In the bottom panels of Figure 7 the averages of the simulations are shown. The average of our simulations evidences a major expression of protein 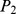 against 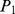, for both values of 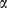, with dumped oscillations for 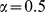 and almost persistent oscillations for 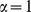.

**Figure 8:**
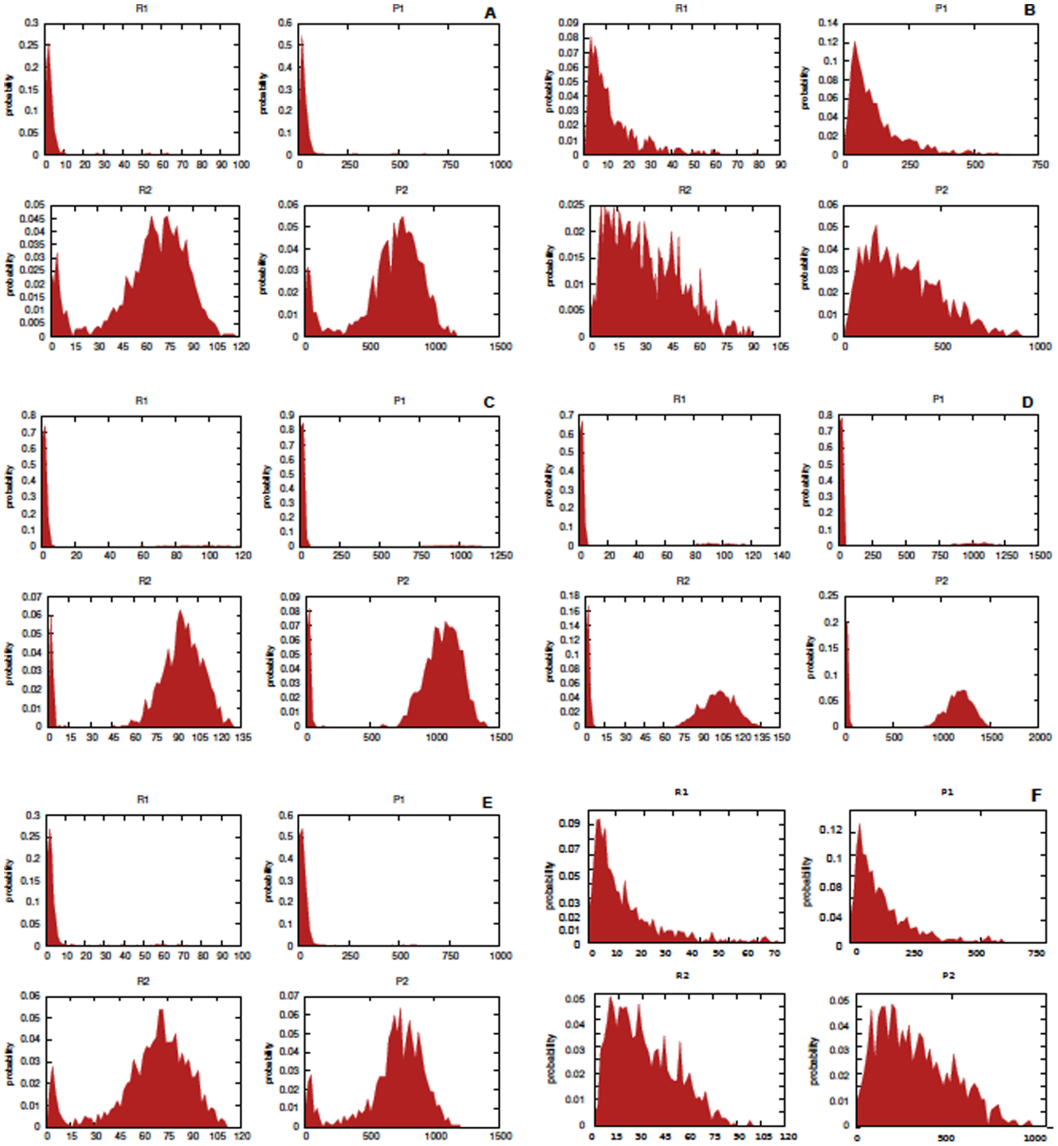
Periodically perturbed toggle switch. Empirical probability density function at various times, after 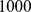 simulations for Zhdanov model with the parameter configurations considered in Figure 7. In **A** 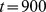 and 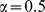, in **B** 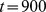 and 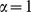, in **C** 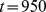 and 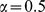, in **D** 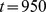 and 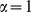, in **E** 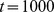 and 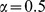 and in **F** 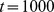 and 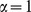.

In Figure 8 we plot the empirical probability density function of the species concentrations, i.e. 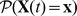 given the considered initial configuration, at 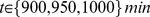 as obtained by 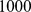 simulations. Interestingly, these bi-modal probability distributions immediately evidence the presence of stochastic bifurcations in the more expressed populations 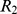 and 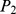. In addition, the distributions for the protein seem to oscillate with period around 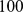, i.e. for 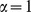 they are unimodal at 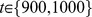 and bi-modal at 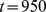.

**Figure 9:**
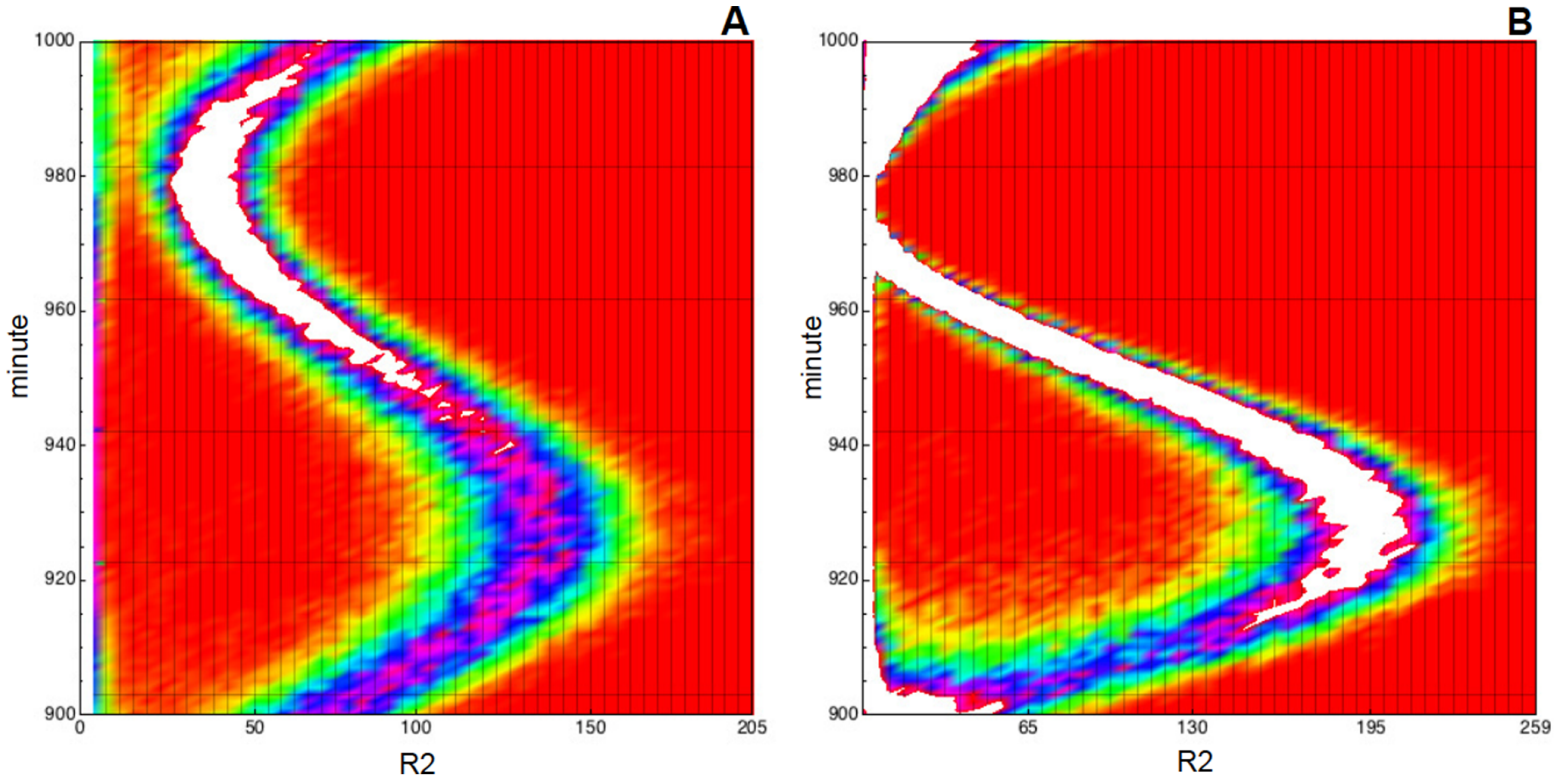
Periodically perturbed toggle switch. Empirical probability density function for 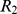 plotted against time, i.e. the probability of being in any reachable state 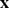 for 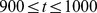. Lighter gradient denotes higher probability values. We used data collected with 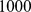 simulations of model (33) where 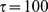 and two perturbation intensities are used, 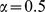 in **A** and 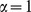 in **B**. In the 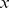-axis the species amount is represented, in the 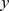-axis the time (in minutes) is given.

For the sake of confirming this hypothesis in Figure 9 the probability density function of 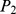 is plotted against time, i.e. the probability of being in state 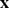 at time 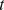, for any reachable state 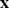 and time 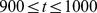. In there we plot a heatmap with time on the 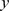-axis and protein concentration on the 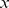-axis; in the figure the lighter gradient denotes higher probability values. Clearly, this figure shows the oscillatory behavior of the probability distributions for both value of 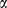 and, more important, explains the uni-modality of the distribution at 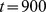 and 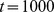 with 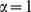, i.e. the higher variance of the rightmost peak at 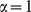 makes the two modes collapse. Finally, we omit to show but, as one should expect, the oscillations of the probability distribution, which are caused by the presence of a sinusoidal perturbation in the parameters, are present and periodic over all the time window 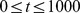.

#### Bounded noises

We investigated the effect of a Sine-Wiener noise affecting protein synthesis rather than a perturbation, i.e. a new 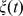 is considered
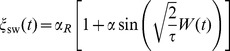
with 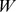 a Wiener process. One the one hand we compared the periodic perturbation proposed by Zhdanov with the sine-Wiener noise because they share three important features: 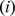 the finite amplitude of the perturbation, 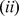 a well-defined time-scale (the period for the sinusoidal perturbation, and the autocorrelation time for the bounded noise), 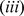 the sinusoidal nature (in one case the sinus is applied to a linear function of time, in the other case is applied to a random walk). On the other hand, especially in control and radio engineering, sinusoidal perturbations are a classical mean to represent external bounded disturbances.

Here simulations are performed by using the SSAn where the reactions in Table 6 are left unchanged, and the propensity functions 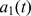 and 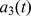 are modified to
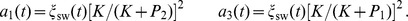



**Figure 10:**
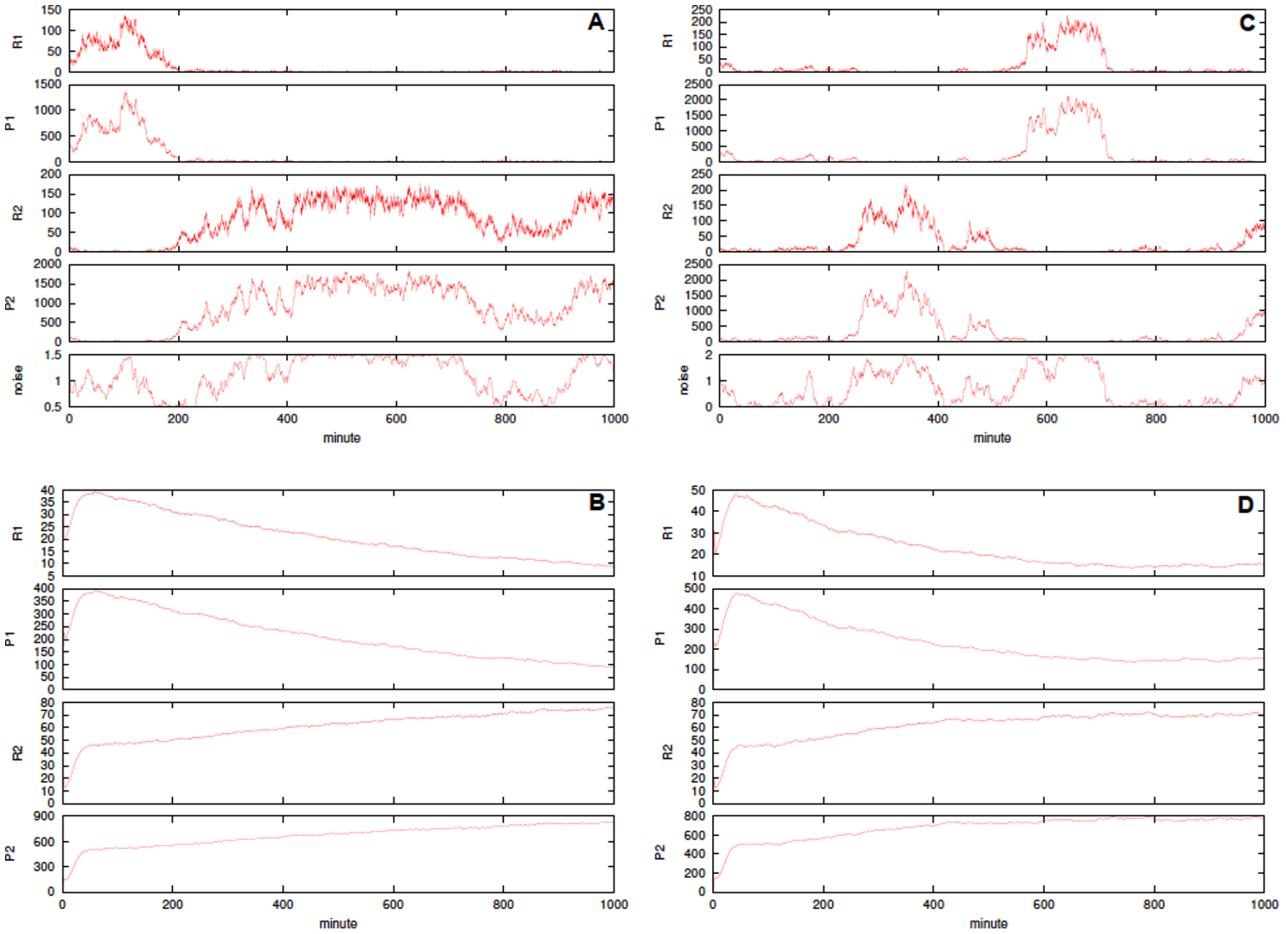
Stochastically perturbed toggle switch. In top plots, single runs for Zhdanov model with Sine-Wiener bounded noise: 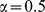 in **A** and 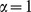 in **C**. In bottom panels the averages of 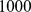 simulations: 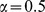 in **C** and 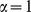 in **D**. In all cases 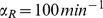, 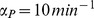, 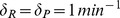, 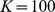 and 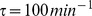 and the initial configuration is 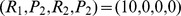. We remark that noise parameters are equivalent to the perturbation of Figure 7; noise realization is plotted for the single runs.

For the sake of comparing the simulations with those in Figures 7, 8, 9, we used the same initial condition and the same values for 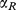, 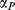, 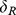, 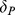 and 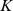. To make reasonable to compare the effect of a realistic noise against the original perturbation we simulated the system with the same values as required, i.e. the noise intensity 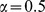 in left and 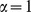 in right of the top panels in Figure 10, and in both cases 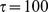. As expected, in this case the trajectories are more scattered than those in Figure 7, and the switches are still present. However, for maximum noise intensity 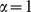 time-slots emerge where the stochastic systems predicts a more complex outcome of the interaction. In fact, for 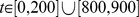 neither protein 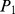 nor 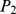 seem to be as expressed as in the other portions of the simulation, thus suggesting the presence of noise-induced equilibria absent when periodic perturbations are present.

**Figure 11:**
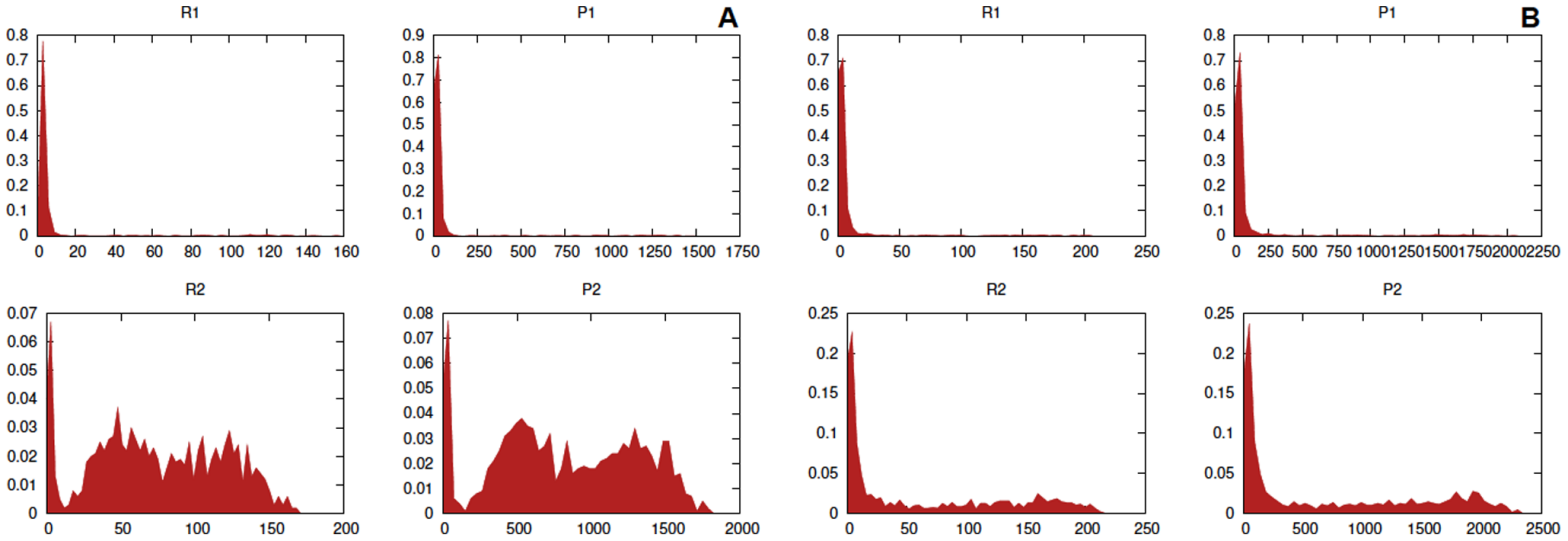
Stochastically perturbed toggle switch. Empirical probability density function at 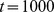, after 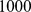 simulations for Zhdanov model with Sine-Wiener noise. Parameters are as in Figure 10 and two perturbation intensities are used: 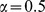 in **A** and 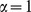 in **B**.

**Figure 12:**
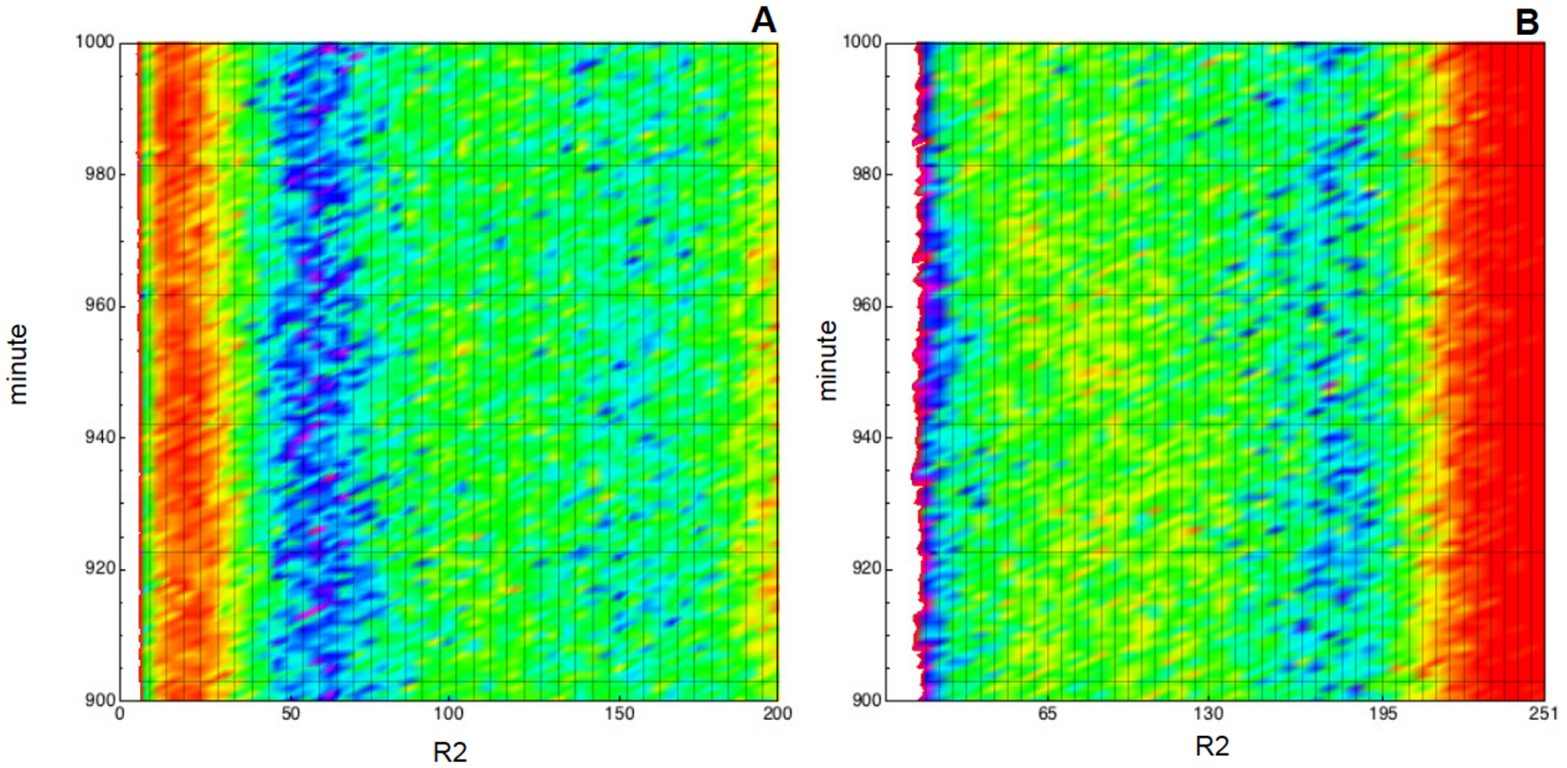
Stochastically perturbed toggle switch. Empirical probability density function for 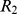 plotted against time, i.e. the DCKE solution for 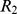 in 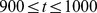. Lighter gradient denotes higher probability values. We used data collected with 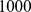 simulations of Zhdanov model with Sine-Wiener noise where 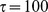 and two perturbation intensities are used: 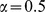 in **A** and 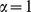 in **B**. In the 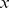-axis the species concentration is represented, in the 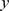-axis minutes are given.

To investigate more in-depth this hypothesis we again performed 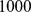 simulations for both the configurations, the averages of which are shown in the bottom panels of Figure 10. In this case, the simulation times, which again depend on the noise correlation, span in range from 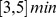 to 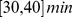, thus making the choice of good parameters crucial. Differently from the case in which a sinusoidal perturbation is considered, i.e. Figure 7, in this case the averages are not oscillatory, but instead show a stable convergence. Also, the final outcome seems again to predict the expression of 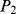 inhibiting 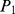. To understand better this point we plotted in Figure 11 the probability density of reachable states at 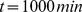, i.e. 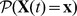 given the considered initial configuration, and in Figure 12 we plotted that distribution against time for 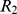. It is worth noting that we also ranged 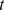 over 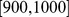 but since 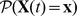 did not change we omitted to plot it here. Again, Figure 12 is a heatmap where on the 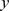-axis time in minutes is given, on the 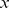-axis the possible concentration for 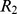 and the lighter gradient denotes higher probability values. Notice that in this case Figure 12 represents an empirical evaluation of the solution the DCKE for this system, i.e. equation (14). Both graphics are obtained by 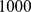 simulations with 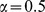 (left panels) and 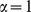 (right panels). These figures show that a low-intensity noise makes the probability distribution become three-modal, i.e. notice the two rightmost peaks in Figure 11 and the white/light-blue gradients in Figure 12. Differently, when the noise intensity is higher, the two rightmost peaks almost merge, thus forming a bi-modal distribution where the smaller peak almost spreads uniformly on the state space for the variables. Notice that, in this case, the amplitude of such a peak is higher than for 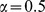, i.e. notice the intensity of the blue gradient in Figure 12. For 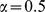 it is possible to notice two red gradients: one approximatively for 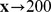 and one for 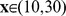. The major peaks in the distribution for 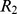 are for 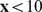, for 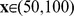 and for 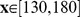. The probability of each of these peaks is decreasing as 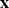 increases, thus confirming the intuition of Figure 11. Similar considerations can be done when 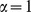 where, as shown by Figure 11, the first dark-red area separating the first two peaks in 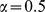 is vanished, thus forming a bi-modal instead that a three-modal probability distribution.

**Figure 13:**
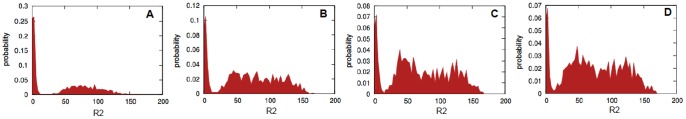
Stochastically perturbed toggle switch. Empirical probability density function at 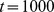 for 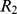, after 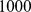 simulations for Zhdanov model with Sine-Wiener noise. In **A** 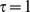, in **B** 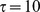, in **C** 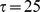 and in **D** 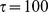. In all cases 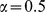 and other parameters are as in Figure 10.

**Figure 14:**
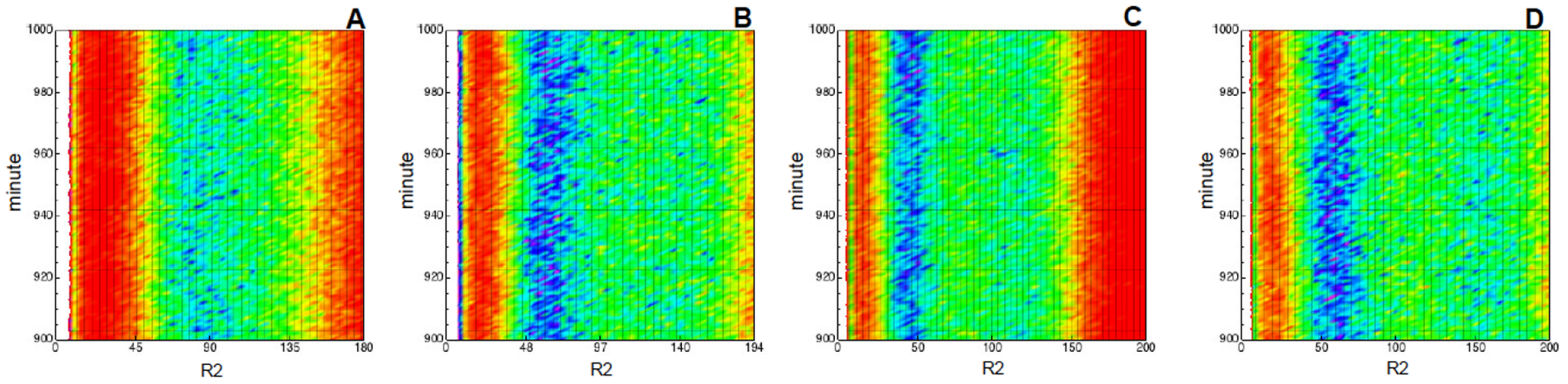
Stochastically perturbed toggle switch. Empirical pro bability density function for 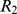 plotted against time, i.e. the DCKE solution for 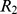 in 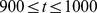. Lighter gradient denotes higher probability values. We used data collected with 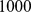 simulations of Zhdanov model with Sine-Wiener noise. In **A** 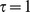, in **B** 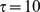, in **C** 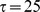 and in **D** 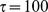. In all cases the noise intensity is 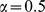. In the 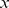-axis the species concentration is represented, in the 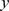-axis minutes are given.

Finally, for the sake of considering a wide range of biologically meaningful values for 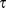, which we recall it represents a measure of the speed of noise variation, we evaluated the solution of the DCKE for 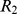 for the same configuration used in Figure 12 and 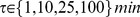. We performed 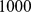 simulations of the model for each value of 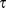 with 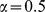, the value showing a more interesting behavior. In Figure 13 the probability of the reachable states at 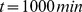 is plotted. If is immediate to notice that the height of the first peak increases as 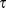 decreases, and more precisely the distribution seems to switch from a three-modal one to a bi-modal when 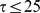. In each panel of Figure 14 we plot the variation of such probability distribution for 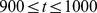. By that figure it is possible to observe that by ranging 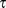 the dark-red gradient increases in size as far as 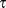 decreases. This means that the amplitude between the peaks of the density strictly depends on the value of 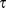, thus suggesting a strong role for extrinsic noise in determining the network functionalities.

## Discussion

In this paper we investigated the effects of joint extrinsic and intrinsic randomness in nonlinear genetic and other biomolecular networks, under the assumption of non-Gaussian bounded external perturbations. As we briefly mentioned in the introduction, the possible impact of bounded extrinsic noise on stochastic biomolecular networks might be manifold, so that this work has to be understood as a first step in this field of investigation. Our applications have shown that the combination of both intrinsic and extrinsic noise-related phenomena may have a constructive functional role also when the extrinsic noise is bounded. This is in line with other researches – only focusing on either intrinsic or extrinsic noise – recasting the classical interpretation of noise as a disturbance more or less obfuscating the real behavior of a network.

This work required the combination of two well-known frameworks, often used to separately describe biological systems. We combined the theory of stochastic chemically reacting systems developed by Gillespie with Langevin systems describing the bounded variations of kinetic parameters. The former shall allow considering the inherent stochastic fluctuations of small numbers of interacting entities, often called intrinsic noise, and clearly opposed to classical deterministic models based on differential equations. The latter permits to consider the influence of bounded extrinsic noises. These noises are modeled as stochastic differential equations. For these kind of systems, although an analytical characterization is unlikely to be feasible, we were able to derive a differential Chapman-Kolgomorov equation (DCKE) describing the probability of the system to occupy each one of a set of states. Then, in order to analyze these models by sampling from this equation we defined an extension of the Gillespie's Stochastic Simulation Algorithm (SSA) with a state-dependent Langevin system affecting the model jump rates. This algorithm, despite being more costly than the classical Gillespie's SSA, allows for the exact simulation of these doubly stochastic systems.

We outlined the role of bounded extrinsic noise for some biological networks of interest. In particular, we were able to extend classical results on the validity of the Michaelis-Menten approximation to the prototypical Enzyme-Substrate-Product enzymatic reaction by drawing a Stochastic Quasi Steady State Assumption (SQSSA) for noisy reactions. Along the line of the classical deterministic or stochastic uses of the Michaelis-Menten approximation, this should permit to reduce the size of more general enzymatic networks even in presence of extrinsic bounded noises.

Moreover, we showed that in a recurrent pattern of genetic and enzymatic networks, i.e. the futile cycle, the presence of extrinsic noises induces the switching from a unimodal probability density (in absence of external perturbations) to a multimodal density.

Similarly, in the case of the toggle switch, which is inherently multistable, the presence of extrinsic noise significantly modulates the probability density of the genes concentration. In this important network motif we also investigated the role of periodic perturbations against a realistic noise.

Thus in general the co-presence of both intrinsic stochasticity and bounded extrinsic random perturbations might suggest the presence of possibly unknown functional roles for noise for these and other networks. The described noise-induced phenomena are shown to be strongly related to physical characteristics of the extrinsic noise such as the noise amplitude and its autocorrelation time.

A relevant issue that we are going to investigate in the next future is the role of the specific extrinsic bounded perturbations. Indeed, in other biological and non-biological systems affected by bounded noises it has been shown that the effects of the perturbations depend not only on the above general characteristics of the noise, but also on its whole model [51], [52], [54], [88]. In other words the transitions of a system perturbed by a sine-Wiener noise might be quite different from those induced by another bounded perturbation, for example the Cai-Lin noise [89] or the Tsallis noise [55], also when their amplitude and autocorrelation times are equal. Thus, a single biomolecular network in two different environments might show two different behaviors depending of fine details of the kind of perturbations that are present. This might also suggest that a same network might exhibit many different functions depending on its “locations”.

Concerning these points, we stress that these peculiar properties of bounded extrinsic perturbations make it even more important the investigations, such as those of [48], aimed at inferring by deconvolution the external noise from the experimental data, in order to infer which kind of noise affect a given network in a well determined environment.

An explicit formalization of biomolecular networks by means of graph-theory and network topology-based analysis of response is outside of our scope and it is not strictly needed for the description and application of our algorithms. However, we want to outline here two important problems in this area, recently considered [90], [91] in the framework of traditional approaches to unbounded extrinsic noises, that deserve future investigations. The first [90] is the evaluation of the relationships between network topologies and robustness to bounded stochastic perturbations or, conversely, ability of exploiting them. The second one [91] is even more important: given a large biomolecular network endowed by nontrivial emergent properties, can the presence of bounded extrinsic noise “constructively” induce new emergent properties?

Finally, note that the methodologies introduced in this work can be applied, virtually without any formal modifications, to a wide range of problems in computational biology of human, animal and cellular populations. Indeed – since the Ross model of malaria spread in 1911 [92], [93], and the prey-predators models by Volterra [94] and Lotka [95] (himself a chemical physicist) – theoretical population biology has successfully adopted the paradigm of the law of mass-action to describe the interplays between subjects in a population [57]. Thus, we are also working in this direction.

## References

[1] Tomas R, d'Ari R (1990) Biological Feedbacks. Chapman & Hall/CRC Mathematical & Computational Biology.

[2] Iglesias PA, Ingalls PB (2010) Control Theory and Systems Biology. MIT Press.

[3] Junker BJ, Schreiber F (eds) (2008) Analysis of Biological Networks. Wiley – Interscience.

[4] Chen L, Wang R-R, Zhang X-S (2009) Biomolecular Networks. Wiley.

[5] Paulsson BO (2011) Systems Biology Simulation of Dynamic Network States. Cambridge University Press.

[6] Yamada T, Bork P (2009) Evolution of biomolecular networks – lessons from metabolic and protein interactions. Nat Rev Mol Cell Bio 10: 791–803. 19851337 10.1038/nrm2787

[7] Alon U (2006) An Introduction to Systems Biology: Design Principles of Biological Circuits. Chapman & Hall/CRC Mathematical & Computational Biology.

[8] Wilkinson U (2006) Stochastic Modelling for Systems Biology. Chapman & Hall/CRC Mathematical & Computational Biology.

[9] Rigney DR, Schieve WC (1977) Stochastic model of linear, continuous protein – synthesis in bacterial populations. J Th Bio 69: 761–766. 10.1016/0022-5193(77)90381-2 607033

[10] Rigney DR (1979) Stochastic models of cellular variability. In R. Thomas (ed.) “Kinetic logic – a Boolean approach to the analysis of complex regulatory systems”. Berlin: Springer – Verlag.

[11] Kauffman SA (1969) Metabolic stability and epigenesis in randomly constructed genetic nets J Th Bio. 22: 437–467. 10.1016/0022-5193(69)90015-0 5803332

[12] Glass L, Kauffman SA (1968) Logical analysis of systems comprising feedback loops. J Th Bio 39: 103–129.

[13] Griffith JS (1968) Mathematics of Cellular Control Processes. II. Positive feedback to One Gene. J Th Bio 20: 209–216. 10.1016/0022-5193(68)90190-2 5727240

[14] Simon Z (1965) Multi – steady – state model for cell differentiation. J Th Biol 8: 258–263. 10.1016/0022-5193(65)90076-7 5876239

[15] Thomas R (1978) Logical analysis of systems comprising feedback loops. J Th Biol 73: 631–656. 10.1016/0022-5193(78)90127-3 703339

[16] Sugita M (1964) Functional analysis of chemical systems in vivo using a logical circuit equivalent. II. The idea of a molecular automaton J Th Bio 4: 437–467. 13918223

[17] Angeli D, Ferrell JE, Sontag ED (2004) Detection of multistability, bifurcations, and hysteresis in a large class of biological positive – feedback systems. Proc Nat Acad Sci US 101 (7): 1822–1827. 10.1073/pnas.0308265100 PMC357011 14766974

[18] Gardner TR, Cantor CR, Collins JJ (2000) Construction of a genetic toggle switch in Escherichiacoli. Nature 403: 339–342. 10659857 10.1038/35002131

[19] Kramer BP, Fussenegger M (2005) Hysteresis in a synthetic mammalian gene network. Proc Nat Acad Sci US 102: 9517–9522. 10.1073/pnas.0500345102 PMC1172236 15972812

[20] Markevich NI, Hoek JB, Kholodenko BN (2004) Signaling switches and bistability arising from multisite phosphorylation in protein kinase cascades. J Cell Bio 164: 353–359. 14744999 10.1083/jcb.200308060 PMC2172246

[21] Siegal–Gaskins D, Grotewold E, Smith GD (2009) The capacity for multistability in small gene regulatory networks. BMC Sys Bio 3:96. Available: http://www.biomedcentral.com/1752-0509/3/96. Accessed 9 January 2013. 10.1186/1752-0509-3-96 PMC2759935 19772572

[22] Wang L, Walker BL, Iannaccone S, Bhatt D, Kennedy PJ, et al (2009) Bistable switches control memory and plasticity in cellular differentiation. Proc Nat Acad Sci US 106 (16): 6638–6643. 10.1073/pnas.0806137106 PMC2672527 19366677

[23] Xiong W, Ferrell JE (2003) A positive – feedback – based bistable ‘memory module’ that governs a cell fate decision. Nature 426: 460–465. 14647386 10.1038/nature02089

[24] Zhdanov VP (2012) Periodic perturbation of genetic oscillations. Chaos Solitons & Fract 45: 577–587.

[25] Zhdanov VP (2009) Interplay of bistable kinetics of gene expression during cellular growth. J Phys A: Math. Theor 42: 065102.

[26] Detwiler PB, Ramanathan S, Sengupta A, Shraiman BI (2000) Engineering aspects of enzymatic signal transduction: photoreceptors in the retina. Biophys J 79: 2801–2817. 11106590 10.1016/S0006-3495(00)76519-2 PMC1301161

[27] Rao CV, Wolf D, Arkin AP (2002) Control, exploitation and tolerance of intracellular noise Nature. 420: 231–237. 10.1038/nature01258 12432408

[28] Becskei A, Serrano L (2000) Engineering stability in gene networks by autoregulation. Nature 405: 590–593. 10850721 10.1038/35014651

[29] Thattai M, Van Oudenaarden A (2001) Attenuation of noise in ultrasensitive signaling cascades. Biophys J 82: 2943–2950. 10.1016/S0006-3495(02)75635-X PMC1302082 12023217

[30] Lestas I, Vinnicombe G, Paulsson J (2010) Fundamental limits on the suppression of molecular fluctuations. Nature 467: 174–178. 20829788 10.1038/nature09333 PMC2996232

[31] Horsthemke W, Lefever R (1984) Noise – Induced Transitions: Theory and Applications in Physics, Chemistry, and Biology. Springer.

[32] Hasty J, Pradines J, Dolnik M, Collins JJ (2000) Noise – based switches and amplifiers for gene expression. Proc Nat Acad Sci US 97 (5): 2075–2080. 10.1073/pnas.040411297 PMC15756 10681449

[33] Samoilov M, Plyasunov S, Arkin AP (2005) Stochastic amplification and signaling in enzymatic futile cycles through noise – induced bistability with oscillations. Proc Nat Acad Sci US 102 (7): 2310–2315. 10.1073/pnas.0406841102 PMC548975 15701703

[34] Becskei A, Kaufmann BB, van Oudenaarden AE (2000) Contributions of low molecule number and chromosomal positioning to stochastic gene expression. Nature Gen 37: 937–944. 10.1038/ng1616 16086016

[35] Elowitz MB, Levine AJ, Siggia ED, Swain PS (2002) Stochastic Gene Expression in a Single Cell. Science 298: 1183–1186. 10.1126/science.1070919 12183631

[36] Ghaemmaghami S, Huh W, Bower K, Howson RW, Belle A, et al (2003) Global analysis of protein expression in yeast. Nature 425: 737–743. 14562106 10.1038/nature02046

[37] Cai L, Friedman N, Xie XS (2006) Stochastic protein expression in individual cells at the single molecule level. Nature 440: 358–362. 16541077 10.1038/nature04599

[38] Gillespie DT (1976) A General Method for Numerically Simulating the Stochastic Time Evolution of Coupled Chemical Reactions. J Comp Phys 22 (4): 403–434.

[39] Gillespie DT (1977) Exact Stochastic Simulation of Coupled Chemical Reactions. J Phys Chem 81: 2340–2361.

[40] Thattai M, Van Oudenaarden A (2001) Intrisic noise in Gene Regulatory Networks. Proc Nat Acad Sci US 98: 8614–8619. 10.1073/pnas.151588598 PMC37484 11438714

[41] Tze–Leung T, Mahesci N (2010) Stochasticity and Cell Fate. Science 327: 1142–1145.20185727

[42] Gardiner CW (1985) Handbook of Stochastic Methods (2nd edition). Springer.

[43] Gillespie DT (1980) Approximating the master equation by Fokker – Planck – type equations for single – variable chemical systems. J Phys Chem 72: 5363–5371.

[44] Grabert H, Hänggi P, Oppenheim I (1983) Fluctuations in Reversible Chemical Reactions Physica A. 117: 300–316.

[45] Gillespie DT (2000) The chemical Langevin equation. J Phys Chem 113: 297–306.

[46] Eldar A, Elowitz MB (2010) Functional role for noise in genetic circuits, Nature. 467: 167–173. 10.1038/nature09326 PMC4100692 20829787

[47] Losick R, Desplan C (2008) Stochasticity and Cell Fate. Science 320: 65–68. 18388284 10.1126/science.1147888 PMC2605794

[48] Hallen M, Li B, Tanouchi Y, Tan C, West M, et al (2011) Computation of Steady – State Probability Distributions in Stochastic Models of Cellular Networks. PLoS Comp Bio 7(10): e1002209. 10.1371/journal.pcbi.1002209 PMC3192818 22022252

[49] Hilfinger A, Paulsson J (2011) Separating intrinsic from extrinsic fluctuations in dynamic biological systems. Proc Nat Acad Sci US 108: 12167–12172. 10.1073/pnas.1018832108 PMC3141918 21730172

[50] d'Onofrio A, editor (in press). Bounded Stochastic Processes in Physics, Biology, and Engineeering. Birkhauser, Boston.

[51] d'Onofrio A (2010) Bounded – noise – induced transitions in a tumor – immune system interplay. Phys Rev E 81: 021923. 10.1103/PhysRevE.81.021923 20365611

[52] d'Onofrio A, Gandolfi A (2010) Resistance to antitumor chemotherapy due to bounded–noise–induced transitions Phys Rev E. 82: 061901. 10.1103/PhysRevE.82.061901 21230684

[53] Bobryk RV, Chrzeszczyk A (2005) Transitions induced by bounded noise. Physica A 358: 263–272.

[54] de Franciscis S, d'Onofrio A (2012) Spatiotemporal Bounded Noises, and transitions induced by them in Ginzburg – Landau model. Phys Rev E 86: 021118. 10.1103/PhysRevE.86.021118 23005733

[55] Wio HR, Toral R (2004) Effect of non – Gaussian noise sources in a noise – induced transition. Physica D 193: 161–168.

[56] Ullah M, Wolkhenauer O (2011) Stochastic Approaches for Systems Biology, Springer.

[57] Murray JD (2002) Mathematical Biology. Springer 3rd edition.

[58] Sanft KR, Gillespie DT, Petzold LR (2011) Legitimacy of the stochastic Michaelis – Menten approximation. IET Sys Bio 5 (1): 58–69. 10.1049/iet-syb.2009.0057 21261403

[59] NoisySIM, 2012. Available: http://sites.google.com/site/giuliocaravagna/. Accessed 2013 January 9.

[60] Doob JL (1942) Topics in the Theory of Markoff Chains. Trans Am Math Soc 52 (1): 37–64.

[61] Doob JL (1945) Markoff chains – Denumerable case. Trans Am Math Soc 58 (3): 455–473.

[62] Gillespie DT, Petzold LR (2006) Numerical Simulation for Biochemical Kinetics. In: Zoltan Szallasi, Jorg Stelling, Vipul Periwa, editors. System modeling in cell biology: from concepts to nuts and bolts, MIT Press. 331–353.

[63] Kolmogorov A (1931) Uber die analytischen Methoden in der Wahrscheinlichkeitsrechnung. Math Ann 104 (1): 415–458.

[64] Mateescu M, Wolf V, Didier F, Henzinger TA (2010) Fast adaptive uniformisation of the chemical master equation. IET Sys Bio 4 (6): 441–452. 10.1049/iet-syb.2010.0005 21073242

[65] Gibson MA, Bruck J (2000) Efficient Exact Stochastic Simulation of Chemical Systems with Many Species and Many Channels. J Phys Chem A 104 (9): 1876–1889.

[66] Cao Y, Gillespie DT, Petzold LR (2005) The Slow – scale Stochastic Simulation Algorithm. J Chem Phys 122 (1): 014116. 10.1063/1.1824902 15638651

[67] Gillespie DT (2001) Approximated Accelerated Stochastic Simulation of Chemically Reacting Systems. J Chem Phys 115 (4): 1716–1733.

[68] Feller W (1940) On the Integro – Differential Equations of Purely Discontinous Markoff Processes. Trans Am Math Soc 48 (3): 4885–15.

[69] Anderson DF (2007) A modified next reaction method for simulating chemical systems with time dependent propensities and delays. J Chem Phys 127: 214107. 18067349 10.1063/1.2799998

[70] Alfonsi A, Cances E, Turinici G, Di Ventura B, Huisinga W (2005) Adaptive simulation of hybrid stochastic and deterministic models for biochemical systems. ESAIM Proc 14: 1–13.

[71] Alfonsi A, Cances E, Turinici G, Di Ventura B, Huisinga W (2004) Exact simulation of hybrid stochastic and deterministic models for biochemical systems. INRIA Tech. Report 5435. Available: http://hal.inria.fr/inria-00070572. Accessed 2013 January 9.

[72] Caravagna G, d'Onofrio A, Milazzo P, Barbuti R (2010) Antitumor Immune Surveillance Through Stochastic Oscillations. J Th Bio 265 (3), 336–345. 10.1016/j.jtbi.2010.05.013 20580640

[73] Caravagna G, Barbuti R, d'Onofrio A (2012) Fine – tuning anti – tumor immunotherapies via stochastic simulations. BMC Bioinf (Suppl 4): S8. 10.1186/1471-2105-13-S4-S8 PMC3303725 22536975

[74] Cox DR (1955) Some Statistical Methods Connected with Series of Events. J Royal Stat Soc 17 (2): 129–164.

[75] Bouzas PR, Ruiz–Fuentes N, Ocaña FM (2007) Functional approach to the random mean of a compound Cox process. Comp Stat 22: 467–479.

[76] Daley D J, Vere–Jones D (2003) An Introduction to the Theory of Point Processes, volume I: Elementary Theory and Methods of Probability and its Applications. Springer, 2nd edition.

[77] Todorovic P (1992) An Introduction to Stochastic Processes and Their Applications. Springer Series in Statistics. Springer.

[78] Stratonovich RL (1963) Topics in the Theory of Random Noise, vol. 1. Gordon and Breach Science Publisher, New York.

[79] Segel LA, Slemrod M (1989) The quasi – steady – state assumption: a case study in perturbation. SIAM Rev 31: 446–477.

[80] Bena I (2006) Dichotomous Markov noise: Exact results for out – of – equilibrium systems. A review. Int J Mod Phys B 20: 2825–2888.

[81] Voet D, Voet JG, Pratt CW (1999) Foundamentals of Biochemistry. Wiley, New York.

[82] Ferrell JE, Machleder EM (1998) The Biochemical Basis of an All – or – None Cell Fate Switch in Xenopus Oocytes. Science 8: 895–898. 10.1126/science.280.5365.895 9572732

[83] Chang HH, Oh PY, Ingber DE, Huang S (2006) Multistable and multistep dynamics in neutrophil differentiation. BMC Cell Bio 7. 10.1186/1471-2121-7-11 PMC1409771 16507101

[84] Cherry JL, Adler FR (2000) How to make a biological switch. J Th Bio 203: 117–130. 10.1006/jtbi.2000.1068 10704297

[85] Cinquin O, Demongeot J (2005) High – dimensional switches and the modelling of cellular differentiation. J Th Bio 233: 391–411. 10.1016/j.jtbi.2004.10.027 15652148

[86] Zhdanov VP (2011) Periodic perturbation of the bistable kinetics of gene expression. Physica A 390 (1): 57–64.

[87] Kaern M, Elston TC, Blake WJ, Collins JJ (2005) Stochasticity in gene expression: from theories to phenotypes. Nature Rev Gen 6: 451–464. 10.1038/nrg1615 15883588

[88] d'Onofrio A (2012) Multifaceted aspects of the kinetics of immunoevasion from tumor dormancy. In: Heiko Enderling, Nava Almog and Lynn Hlatky, editors. Systems Biology of Tumor Dormancy. Advances in Experimental Medicine and Biology, Vol. 734. Springer Verlag. 111–144.

[89] Cai CQ, Lin YK (1996) Generation of non – Gaussian stationary stochastic processes. Phys Rev E 54: 299–303. 10.1103/physreve.54.299 9965073

[90] Chalancon G, Ravarani CNJ, Balaji S, Martinez–Arias A, Aravind L, et al (2012) Interplay between gene expression noise and regulatory network architecture. Trends Gen 28: 221–232. 10.1016/j.tig.2012.01.006 PMC3340541 22365642

[91] Nacher JC, Ochiai T (2011) Emergent Principles in Gene Expression Dynamics. Open Bioinf J 5: 34–41. Available: http://www.benthamscience.com/open/tobioij/articles/V005/SI0001TOBIOIJ/34TOBIOIJ.htm. Accessed 2013 January 9.

[92] Ross R (1911) Some quantitative studies in epidemiology. Nature 87: 466–467.

[93] Smith DL, Battle KE, Hay SI, Barker CM, Scott TW, et al. (2012) Ross, Macdonald, and a Theory for the Dynamics and Control of Mosquito – Transmitted Pathogens. PLoS Path 8(4): e1002588. Available: http://www.plospathogens.org/article/info%3Adoi%2F10.1371%2Fjournal.ppat.1002588, Accessed 2013 January 9. 10.1371/journal.ppat.1002588 PMC3320609 22496640

[94] Volterra V (1926) Fluctuations in the abundance of a species considered mathematically, Nature. 118: 558–560.

[95] Lotka AJ (1925) Elements of Physical Biology, Baltimore: William & Wilkins Company.

